# Cystathionine β-synthase as novel endogenous regulator of lymphangiogenesis via modulating VEGF receptor 2 and 3

**DOI:** 10.1038/s42003-022-03923-7

**Published:** 2022-09-10

**Authors:** Niloofar Hatami, Christian Büttner, Felix Bock, Sara Simfors, Gwen Musial, André Reis, Claus Cursiefen, Thomas Clahsen

**Affiliations:** 1 Department of Ophthalmology, University of Cologne, Faculty of Medicine and University Hospital Cologne, Cologne, Germany; 2 Institute of Human Genetics, University Hospital Erlangen, Friedrich-Alexander-University Erlangen-Nürnberg, Erlangen, Germany; 3 Center for Molecular Medicine (CMMC), University of Cologne, Cologne, Germany

**Keywords:** Corneal diseases, Lymphangiogenesis

## Abstract

Lymphangiogenesis is a key player in several diseases such as tumor metastasis, obesity, and graft rejection. Endogenous regulation of lymphangiogenesis is only partly understood. Here we use the normally avascular cornea as a model to identify endogenous regulators of lymphangiogenesis. Quantitative trait locus analysis of a large low-lymphangiogenic BALB/cN x high-lymphangiogenic C57BL/6 N intercross and prioritization by whole-transcriptome sequencing identify a novel gene responsible for differences in lymphatic vessel architecture on chromosome 17, the *cystathionine β-synthase (Cbs)*. Inhibition of CBS in lymphatic endothelial cells results in reduce proliferation, migration, altered tube-formation, and decrease expression of vascular endothelial growth factor (VEGF) receptor 2 (VEGF-R2) and VEGF-R3, but not their ligands VEGF-C and VEGF-D. Also in vivo inflammation-induced lymphangiogenesis is significantly reduce in C57BL/6 N mice after pharmacological inhibition of CBS. The results confirm CBS as a novel endogenous regulator of lymphangiogenesis acting via VEGF receptor 2 and 3-regulation and open new treatment avenues in diseases associated with pathologic lymphangiogenesis.

## Abstract (web summary)

Quantitative trait locus analysis of BALB/cN x C57BL/6 N intercross identifies cystathionine β-synthase as a previously unknown regulator of lymphangiogenesis by affecting VEGF-R2/VEGF-R3 expression in lymphatic endothelial cells.

## Introduction

Lymphangiogenesis is critically involved in numerous (patho)physiological processes such as tissue fluid regulation, immune surveillance, tumor metastasis, obesity, cardiovascular and neurodegenerative diseases, glaucoma as well as (corneal) graft rejection^1^.

The cornea, the “windscreen of the eye”, under normal conditions is free of both blood and lymphatic vessels^2^. The avascularity of the cornea is a prerequisite for its transparency which is maintained by an active process involving the production of antiangiogenic factors to counterbalance proangiogenic stimuli^3^.

Lymphangiogenesis is mainly induced by binding of the vascular endothelial growth factor (VEGF)-C and VEGF-D to its receptors VEGF-R2 and VEGF-R3^4,5^. In addition, other cytokines have been identified, like the fibroblast growth factor-2 (FGF-2), hepatocyte growth factor (HGF), platelet-derived growth factor (PDGF), insulin-like growth factor (IGF), and both indirectly and directly VEGF-A, which have the capacity to induce lymphangiogenesis^6–8^. Beside the cytokines that promote lymphangiogenesis, there also exist cytokines that have antilymphangiogenic effect, like transforming growth factor (TGF)-β and interferon (IFN)-γ^8–10^.

Endogenous inhibitors play an important regulatory role in preserving corneal angiogenic privilege and inhibiting and regressing lymphatic vessels induced by minor vascular stimuli. Here the corneal epithelium plays a central role. The corneal epithelium expresses both the soluble VEGF-R2 and VEGF-R3 which serve as decoy receptors and trap VEGF-C and VEGF-D^11,12^. The binding of VEGF-C to the sVEGF-R3 not only inhibits lymphangiogenesis, but also causes regression of already formed lymphatic vessels^12,13^. In addition to that, the membrane bound form of the VEGF-R3 is also expressed by corneal epithelial cells and is able to bind VEGF-C, thereby maintaining corneal avascularity^2,3^.

In recent years, only a few novel endogenous modulators of lymphangiogenesis that play an important role in maintaining the angiogenic privilege have been identified. Among these identified and accepted endogenous inhibitors are thrombospondin-1^14,15^, vasohibin-1^16,17^, MT-MMP1^18^, neuropilin (NP-2)^19^, semaphorin-3F (Sema-3F)^20^, endostatin^21^ and tyrosinase^22^.

The cornea is a well-established and widely used in vivo model to analyze the underlying mechanisms of (lymph)angiogenesis^23^. Advantages of the murine cornea are its physiological avascularity, transparency, and exposed position. Following experimentally induced inflammation, both blood and lymphatic vessels arising from pre-existing ones in the corneal limbus can propagate into the cornea^6,24^. The ingrowth of blood and lymphatic vessels in patients not only leads to reduced vision, but also increases the risk for immune reactions after subsequent corneal transplantation^25^. The analysis of corneal lymphatic vessels in different mouse strains revealed strain-specific differences both in inflammation-induced and VEGF-C-induced lymphangiogenesis as well as in the vasculature of the naive cornea^26^. The results suggest underlying genetic factors influencing the lymphangiogenic response^26^. By performing pathway-specific expression analysis of the cornea of low-lymphangiogenic BALB/cN with high-lymphangiogenic C57BL/6 N mice, two novel endogenous regulators of lymphangiogenesis (TRAIL and PLAT/tPa) have been identified that confer to the lymphangiogenic privilege of the cornea^23^.

Lymphangiogenesis is a quantitative trait which can be determined by measuring multiple distinct morphometric parameters^27^. Recently by using a large BALB/cN x C57BL/6 N intercross for quantitative trait locus (QTL) analysis, we identified the *tyrosinase gene* on chromosome 7 as a candidate responsible for differences in the architecture of limbal lymph vessels. In addition, tyrosinase has been confirmed to be a new regulator of lymphangiogenesis in developmental and inflammatory lymphangiogenesis^22^.

Herein, we further characterize the second quantitative trait locus to identify additional endogenous modulators of lymphangiogenesis responsible for the observed differences in low-lymphangiogenic BALB/cN and high-lymphangiogenic C57BL/6 N mice. We identified CBS as a novel endogenous regulator of lymphangiogenesis primarily affecting the VEGF-R2 and VEGF-R3 expression on lymphatic endothelial cells thus opening new treatment modalities for lymphangiogenesis-related diseases.

## Results

### Identification of Cystathionine β-synthase as a potential new endogenous modulator of lymphangiogenesis by prioritization of QTL genes using differential expression and public data

We previously identified significant difference in limbal lymphatic vasculature of naive corneas between C57BL/6 N and BALB/cN mice (Supplementary Fig. 1)^26^. The naive cornea of the C57BL/6 N mice showed a significantly higher lymphatic surface area compared with BALB/cN mice (Supplementary Fig. 1a, b)^26^. To further characterize the complexity of the lymphatic vessel network, the numbers of sprouts, branching points, and endpoints in the naive cornea of C57BL/6 N and BALB/cN mice were evaluated. The C57BL/6 N mice showed significantly more sprouts, branching points, and endpoints related to the total corneal area (Supplementary Fig. 1c). Recently we have mapped QTLs that influence the difference in lymphangiogenesis between these two strains of mice. For this purpose, we generated an F_2_ intercross population from BALB/cN and C57BL/6 N mice consisting of 873 F_2_ animals^22^. Corneal whole mounts of these F_2_ animals were immunostained for LYVE-1, a marker specific for lymphatic vessels^2^. 795 F_2_-animals were successfully stained and fully phenotyped. Five morphometric parameters, the lymph vessel area, lymph vessel length, the number of branching points, end points, and vascular extensions from the main limbal lymphatic vessel (sprouts) were determined from microscopic images of cornea whole mounts by using the image analysis program cell^F, and related to the corneal region of interest^22^. The data show a unimodal, continuous distribution of all phenotypes in the F2 population that exceeded both the upper and lower range of the parental strains^22^.

Interval mapping on the basis of a genome-wide grid of 200 Taqman-genotyped SNP markers (genotyping rate, 99.4%) found a prominent locus on chromosome 7 shared by all five phenotypes as well as additionally two to six other loci with a strong overlap between phenotypes. The signal on chromosome 7 resulted from genetic differences in the *tyrosinase* gene recently described as novel endogenous regulator of developmental and inflammatory lymphangiogenesis^22^.

**Fig. 1:**
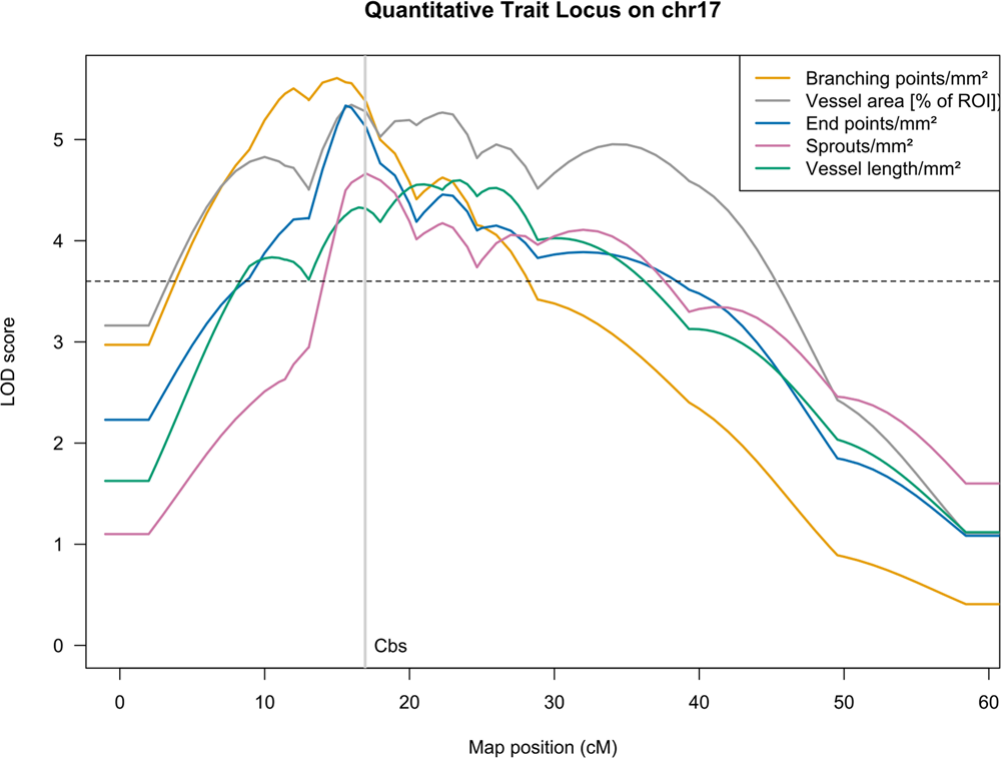
Genome-wide logarithm of the odds (LOD) score profiles showing quantitative trait loci (QTL) for five lymphangiogenesis-related phenotypes. Genome-wide QTL profiles for vessel length/mm^2^ (green), vessel area (percentage; gray), branching points/mm^2^ (orange), end points/mm^2^ (blue), and sprouts/mm^2^ (purple, all Box-Cox-Transform) mapped in 795 F_2_ animals using R/qtl’s stepwise forward selection and backward elimination search for the best multilocus model based on Haley-Knott regression and penalized LOD scores. Loci included in the model are plotted as the result of stepwise QTL; additional chromosomes are added using the addqtl search for a further locus. The threshold for genome-wide significance was determined empirically for each phenotype by 10,000 permutations (maximal autosomal threshold for any phenotype LOD = 3.63 as dashed line). Genome-wide significant loci for all phenotypes have been identified on chromosomes 17.

This analysis further investigates the locus on chromosome 17 (Fig. 1) and also searched for significant genome-wide associations for all five phenotype parameters with a maximum LOD score of 5.61 for branching points per mm² of ROI, spanning a minimum chromosomal interval of chr17:3.36–55.32 Mb (mm10)^22^. This locus needed further refinement and additional assumptions to be useful.

The locus-specific effect plots show a strong additive influence of the C57BL/6 N allele on all five phenotypes at rs13483012, the marker closest to *Cystathionine β-synthase (Cbs)*, consistent with the regulatory role proposed here (Supplementary Fig. 2). The genes contained in the locus on chr17 were further prioritized under the assumption, that the signal might be the effect of an expression difference in cis which would likely be detectable in the parental strains of the mapping population. We therefore sequenced RNA extracted from corneas of mice genetically identical to the F0 animals (Supplementary Table 1). Differential expression analysis on the gene level found 998 genes (Supplementary Data 1) with significant expression differences at a cutoff of *p* ≤ 0.01 (Benjamini–Hochberg adjusted). 489 entities in the Ensembl gene model were upregulated and 509 downregulated in BALB/cN when compared to C57BL/6 N.

Intersecting the set of differentially expressed genes with the locus on chr17 for the phenotype with highest LOD score and smallest 99% Bayesian confidence interval and restricting the analysis to protein-coding or antisense genes (thereby removing pseudogenes and genes still to be experimentally confirmed) the remaining number of genes is 50, 22 of them upregulated and 28 downregulated. 36 of the 50 genes have absolute log-fold-changes > 1, 26 genes > 2 (Table 1).

**Table 1:** Differentially expressed genes after filtering overlapping the quantitative trait locus on chr17.

MGI Symbol	log2FoldChange	*p*-value adjusted (BH)	MGI ID	Ensembl_Gene_IDs
Cbs	−5,16	3,86E-14	MGI:88285	ENSMUSG00000024039
Msln	−4,32	4,13E-03	MGI:1888992	ENSMUSG00000063011
Cdsn	−4,00	3,86E-14	MGI:3505689	ENSMUSG00000039518
H2-DMb1	−2,52	9,10E-03	MGI:95922	ENSMUSG00000079547
Adgrf4	−2,14	3,86E-14	MGI:1925499	ENSMUSG00000023918
Adgrf2	−2,12	6,58E-08	MGI:2182728	ENSMUSG00000057899
Paqr4	−2,01	3,86E-14	MGI:1923748	ENSMUSG00000023909
Hmga1	−2,00	3,86E-14	MGI:96160	ENSMUSG00000046711
Gng13	−1,77	4,57E-03	MGI:1925616	ENSMUSG00000025739
BC051226	−1,68	2,18E-05	MGI:3039585	ENSMUSG00000092564
Dnase1l2	−1,59	9,72E-04	MGI:1913955	ENSMUSG00000024136
Rps2	−1,56	2,53E-07	MGI:105110	ENSMUSG00000044533
Angptl4	−1,11	2,15E-03	MGI:1888999	ENSMUSG00000002289
Acat2	−1,02	5,87E-06	MGI:87871	ENSMUSG00000023832
Atp6v0c	−0,95	3,18E-03	MGI:88116	ENSMUSG00000024121
Ephx3	−0,94	7,00E-06	MGI:1919182	ENSMUSG00000037577
Riok2	−0,91	1,97E-03	MGI:1914295	ENSMUSG00000116564
Amdhd2	−0,87	2,49E-03	MGI:2443978	ENSMUSG00000036820
Slc44a4	−0,82	7,32E-03	MGI:1917379	ENSMUSG00000007034
Wiz	−0,78	2,19E-07	MGI:1332638	ENSMUSG00000024050
Satb1	−0,70	4,51E-03	MGI:105084	ENSMUSG00000023927
Cd2ap	−0,47	1,37E-03	MGI:1330281	ENSMUSG00000061665
Tapbp	0,55	2,69E-04	MGI:1201689	ENSMUSG00000024308
Guca1a	0,56	1,56E-03	MGI:102770	ENSMUSG00000023982
Itpr3	0,56	1,30E-03	MGI:96624	ENSMUSG00000042644
Btbd9	0,56	9,89E-03	MGI:1916625	ENSMUSG00000062202
Mocs1	0,64	2,42E-04	MGI:1928904	ENSMUSG00000064120
H2-T23	0,96	2,45E-04	MGI:95957	ENSMUSG00000067212
Cpne5	1,01	2,84E-04	MGI:2385908	ENSMUSG00000024008
Fgd2	1,01	4,04E-03	MGI:1347084	ENSMUSG00000024013
Ppp1r18	1,02	8,99E-03	MGI:1923698	ENSMUSG00000034595
Glo1	1,08	2,53E-08	MGI:95742	ENSMUSG00000024026
Runx2	1,15	3,52E-06	MGI:99829	ENSMUSG00000039153
Psmb9	1,16	1,02E-03	MGI:1346526	ENSMUSG00000096727
Tap1	1,18	4,62E-09	MGI:98483	ENSMUSG00000037321
Vegfa	1,19	9,43E-04	MGI:103178	ENSMUSG00000023951
Enpp5	1,31	3,86E-14	MGI:1933830	ENSMUSG00000023960
Enpp4	1,33	1,37E-05	MGI:2682634	ENSMUSG00000023961
Clic5	1,44	6,66E-13	MGI:1917912	ENSMUSG00000023959
H2-Q4	1,45	1,09E-13	MGI:95933	ENSMUSG00000035929
A930015D03Rik	1,63	8,83E-04	MGI:1925060	ENSMUSG00000092368
Tmprss3	1,66	2,47E-03	MGI:2155445	ENSMUSG00000024034
Pde9a	2,01	3,86E-14	MGI:1277179	ENSMUSG00000041119
Lst1	2,29	3,45E-11	MGI:1096324	ENSMUSG00000073412
Aif1	2,99	3,86E-14	MGI:1343098	ENSMUSG00000024397
1700031A10Rik	3,94	3,86E-14	MGI:1920536	ENSMUSG00000092239
Apobec2	4,03	4,49E-10	MGI:1343178	ENSMUSG00000040694
Prss41	4,41	9,00E-03	MGI:1918253	ENSMUSG00000024114
H2-T24	4,53	3,86E-14	MGI:95958	ENSMUSG00000053835
Gm11127	8,78	5,10E-06	MGI:3779381	ENSMUSG00000079492

**Fig. 2:**
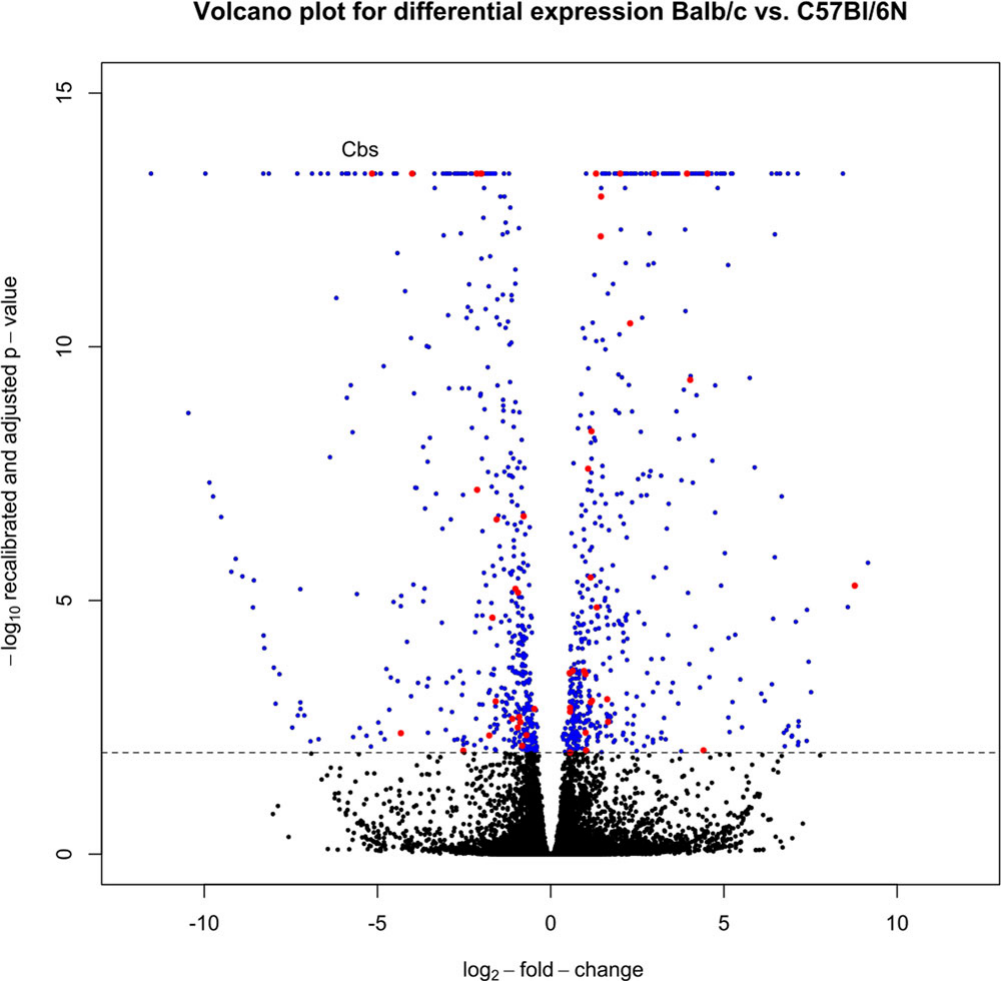
Volcano plot of DESeq2 calculated differential expression results from RNA sequencing. Log_2_-fold-changes comparing BALB/cN to C57BL/6 N expression (x-axis) plotted against -log(10) of the recalibrated p-value after adjustment for multiple testing (*y*-axis). Significantly differentially expressed genes with adjusted *p* < = 0.05 (dotted line) colored blue, non-significant differences in black, differentially expressed genes overlapping chr17 locus after filtering for relevant transcript types colored red, with Cbs as the most strongly downregulated gene after filtering.

*Cbs* is the most highly downregulated QTL gene in BALB/cN with a log2-fold-change of -5.16 compared to C57BL/6 N (adjusted *p*-value = 3.86 × 10^−14^) (Fig. 2). This is equivalent with a de facto expression drop out for *Cbs* in BALB/cN mice corneas. The locus also contains three genes of established importance for vascular development and morphology: *vascular endothelial growth factor A*, *runt related transcription factor 2*, and *angiopoietin-like 4* which show much lower log2-fold-changes of 1.19, 1.15, and −1.11, respectively. *Cbs* itself is close to the center of the LOD interval for all phenotypes and matches the peak of the LOD curve for the phenotype vessel area in % of ROI.

Single nucleotide polymorphisms of various classes are abundant in the region, among them only one homozygous stop gain variant affecting isoforms of *Mocs1*, a C to T exchange at position chr17:49449137. This variant can be found in the majority of Sanger-strains though, with the gene showing annotations of unrelated function. Missense variants have been predicted for at least one transcript of 25 genes, including *Cbs* and *Angpl4*. The missense variant in *Angpl2* is predicted to be tolerated with high confidence by SIFT. For *Cbs* the Sanger mouse genomes variant call set contains 17 variants distinguishing BALB/c from C57BL/6 N, 15 of which are called homozygous. One missense variant at coding sequence position 177 resulting in an Asp to Glu exchange on the protein level according to Ensembl variant prediction is also a potential regulatory variant (rs13482947). In addition, there is one other potential regulatory variant in the promotor- (rs33092461) and two in the promotor-flanking region (rs29515104, rs261335110).

Missense SNPs with non-tolerated predicted consequence (SIFT) were found in *Transporter 1, ATP Binding Cassette Subfamily B Member (Tap1)*, *Major Histocompatibility Complex, Class II, DM Beta (H2-DMb1)*, *Rio Kinase 2 (Riok2)*, *Adhesion G Protein-Coupled Receptor F4 (Adgrf4)*, and *MHC classIb T15 (Gm11127)*. We considered none of these a first-tier candidate with respect to functional annotation in databases and the literature. For *Gm11127*, a gene predicted to be involved in antigen processing and immune response in a large-scale computational annotation effort^28^, is strongly upregulated in BALB/cN mice, though.

None of the locus genes contains a short exonic insertion or deletion and none of the known larger structural variants is a deletion affecting any exon. For *Cbs* the Sanger mouse genomes project contains a 40 bp insertion in intron 7 but the exon structure for both strains is identical with respect to the length of the mean or longest transcript and the total length of all exons. No structural variant could therefore compromise the alignment or counting processes during RNAseq data analysis for *Cbs*.

### CBS inhibition by aminooxyacetic acid effects proliferation, migration, and vessel formation of lymphatic endothelial cells

To functionally characterize a potential role of CBS in lymphangiogenesis, we first studied the in vitro effect of CBS on the complex process of lymphangiogenesis which involves a triad of cellular responses namely proliferation, cell migration, and the organization of lymphatic endothelial cells into new capillary structures. In the search for further previously unknown regulators, we focused on genes of relevant transcript types in which locus information, significant expression differences, and potentially high-impact variants come together. One candidate on chr17 which seemed even more interesting when evaluating the literature for functional clues is Cbs. For Cbs has been described, that it is involved in the regulation of angiogenic parameters^29^.

Therefore, we started investigating whether human CBS influences lymphangiogenesis. We chose a common inhibitor of CBS, aminooxyacetic acid (AOAA) to analyze this in vitro. As the proliferation of lymphatic endothelial cells is the first step in lymphangiogenesis we analyzed the effect of different concentration of AOAA on the proliferation of human dermal lymphatic endothelial cells (HDLECs).

**Fig. 3:**
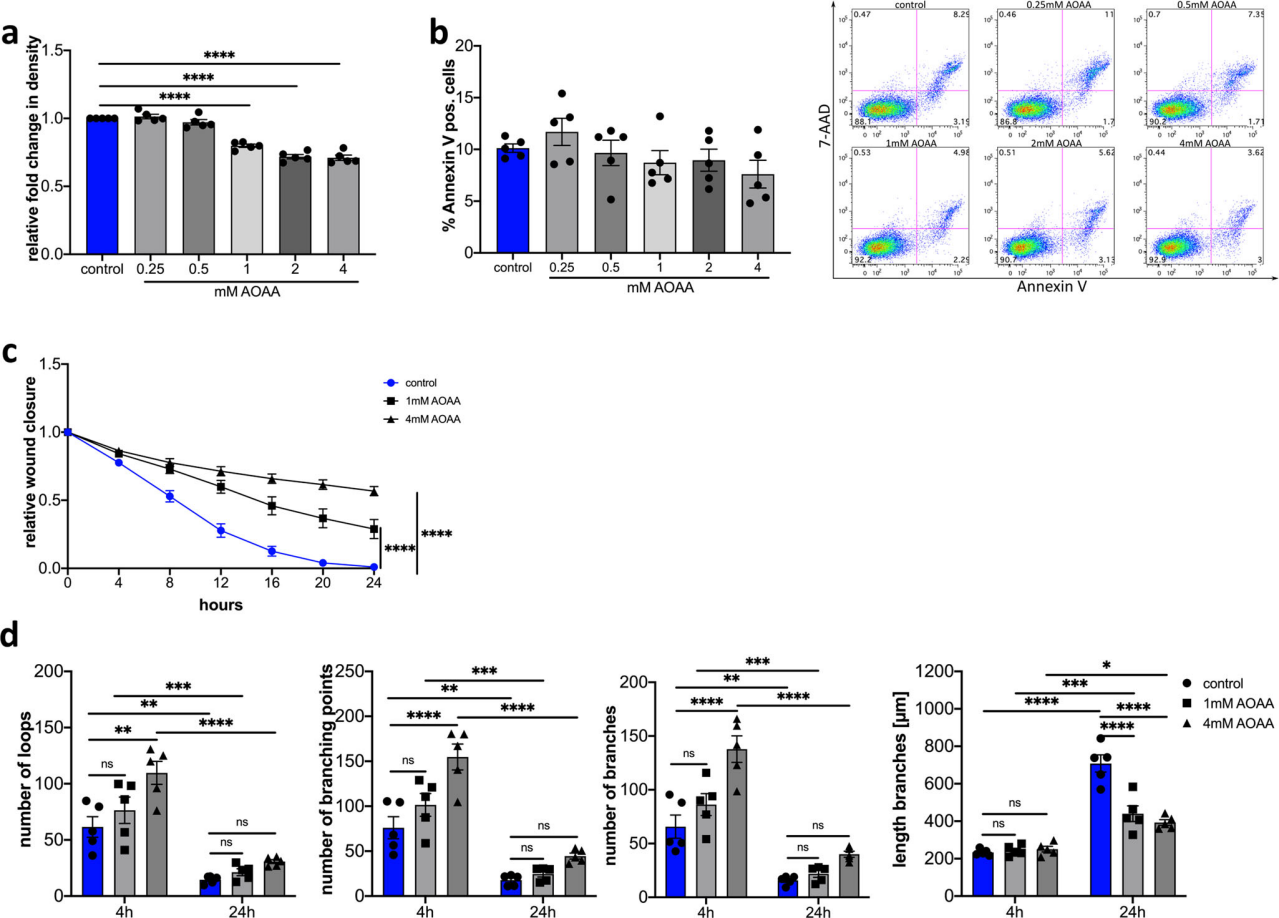
Effect of CBS inhibition by AOAA on HDLEC proliferation, migration and tube formation efficiency. **a** Effect of various doses of AOAA on the proliferation efficiency of HDLECs after 24 h (*n* = 5). Proliferation was determined by using IncuCyte Zoom. **b** Quantification of flow cytometrically detected Annexin V-positive cells 24 h after treatment with the indicated concentrations of AOAA and representative flow cytometry images (*n* = 5). **c** AOAA treatment of HDLECs slowed wound closure compared to control cells in wound healing assay (*n* = 4). **d** Effect of 1 mM and 4 mM AOAA treatment on HDLECs on tube formation. Evaluation was performed after 4 h and 24 h (*n* = 5). Data are presented as means ± SEM. Statistical significance was analyzed with (**a**, **b**) one-way ANOVA and Dunnett’s multiple comparison test or (**c**, **d**) two-way ANOVA and Tukey’s multiple comparison test. *p < 0.05, **p < 0.01; ***p < 0.001; ****p < 0.0001.

Treatment of HDLECs with 0.25 mM and 0.5 mM AOAA showed no influence on proliferation of the cells after 24 h (Fig. 3a). A significant decrease in proliferation of HDLECs was observed 24 h after treatment with 1 mM AOAA compared to control cells (Fig. 3a). Higher concentrations of AOAA (2 mM and 4 mM) lead to a further reduction of proliferation compared to cells treated with 1 mM (Fig. 3a). To be sure that this reduction of proliferation is not caused by increased induction of apoptosis in HDLECs, we determined the apoptotic cells by means of Annexin V staining. Treatment of HDLECs with the used concentrations of AOAA in proliferation assay neither increased the amount of Annexin V positive cells in general nor lead to an increase in necrotic cells (Fig. 3b). There is a tendency that the treatment of HDLECs with 4 mM AOAA has an anti-apoptotic effect (Fig. 3b). Since the treatment with the different concentrations of AOAA did not induce apoptosis, this leads to the question of whether the treatment with AOAA induces cellular senescence. To evaluate this, we treated HDLECs with different concentrations of AOAA for 24 h and perform immunofluorescence staining for cellular senescence marker p16^INK4A^. No significant differences in p16^INK4A^ expression were observed in HDLECs treated with different concentrations of AOAA after 24 h (Supplementary Fig. 3a, b). There is only a trend towards a decreased expression of p16^INK4A^ expression in HDLECs treated with 4 mM AOAA (Supplementary Fig. 3b).

The next step in the lymphangiogenesis process is the migration of the lymphatic endothelial cells. Therefore, a scratch wound assay was carried out to evaluate the influence of CBS on cell migration of lymphatic endothelial cells. The treatment of HDLECs with 1 mM AOAA results in significantly delayed wound closure compared to control. However, treatment of HDLECs with 4 mM AOAA leads to a more pronounced delayed wound closure in comparison to HDLECs treated with 1 mM AOAA as well to control (Fig. 3c).

Thereafter, the migrated cell reorganizes into new capillary structures. We were interested in whether the treatment with AOAA influences the formation of new capillary structures. For this purpose, HDLECs were treated with 1 mM AOAA or 4 mM AOAA and the number of loops, branching points, branches, and the length of branches were determined after 4 h and 24 h. Treatment of HDLECs with 1 mM AOAA did not result in a significant increase in the number of loops, branching points, and branches after 4 h compared to control HDLECs. In contrast, treatment of HDLECs with 4 mM AOAA for 4 h significantly increases the number of loops, the number of branching points, and the number of branches (Fig. 3d). However, neither the treatment of HDLECs with 1 mM AOAA nor with 4 mM AOAA did show any changes in the length of the branches (Fig. 3d). The comparison of 4 h with 24 h clearly shows that over time the number of loops, branching points, and branches decreases, whereas the branches become longer (Fig. 3d).

No significant differences in the number of loops, branching points, and branches were observed 24 h after treatment with either 1 mM or 4 mM AOAA. Here a significant reduction in the length of the tubes could be observed after treatment with either 1 mM AOAA or 4 mM AOAA in comparison to control HDELCs after 24 h (Fig. 3d).

Since the treatment of HDLECS with 4 mM AOAA results in considerable inhibition of proliferation, migration, an altered tube formation capacity, and an anti-apoptotic effect on HDLECS, we used 4 mM AOAA for all other experiments.

### CBS inhibition by aminooxyacetic acid leads to reduced expression of VEGF receptor 2 and 3 by lymphatic vascular endothelial cells

**Fig. 4:**
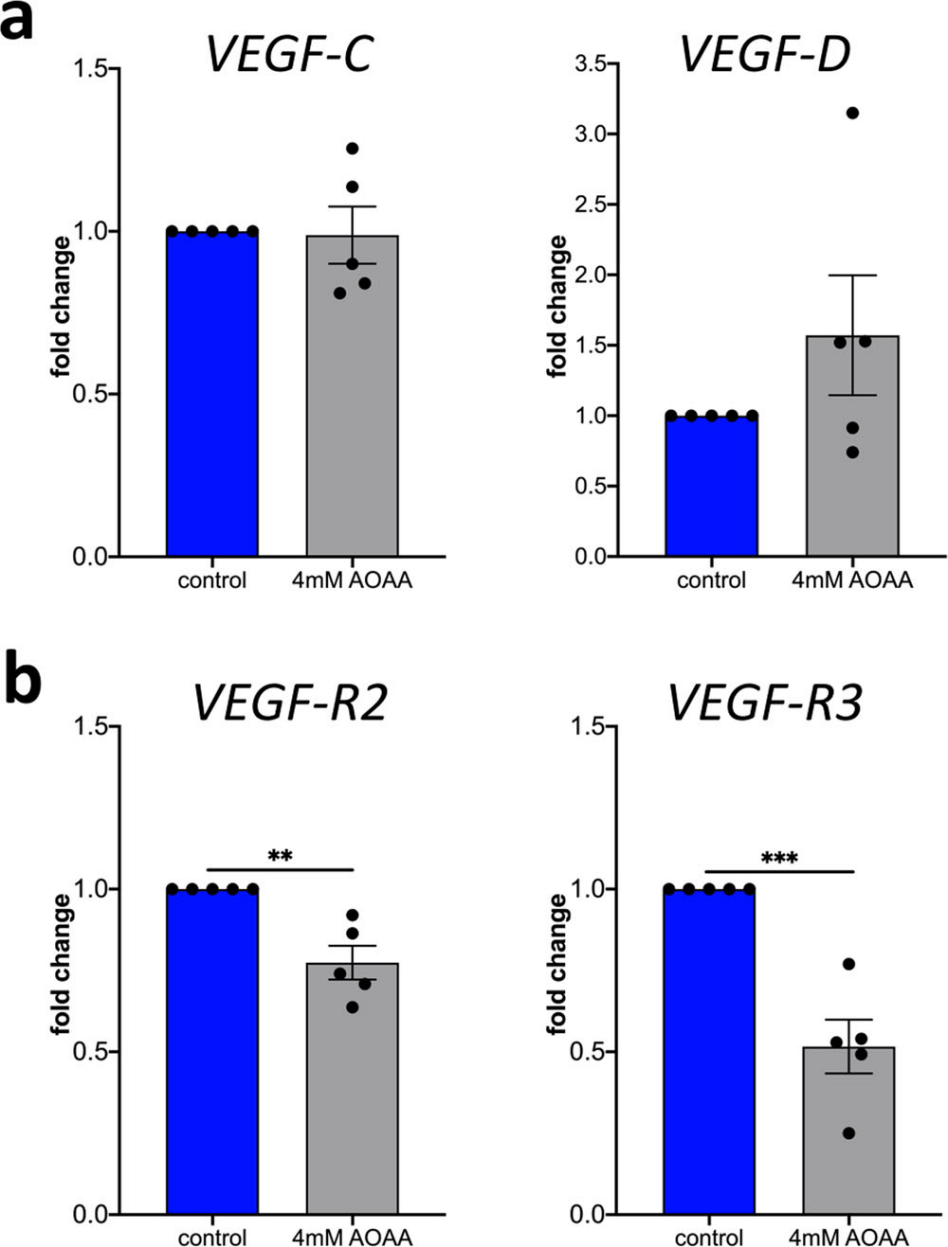
AOAA treatment reduces expression of VEGF-R2 and VEGF-R3 in HDLECs without major effects on VEGF-C and VEGF-D. HDLECs were treated with indicated concentrations of AOAA for 24 h. Level of mRNA for (**a**) *VEGF-C*, and *VEGF-D*, (**b**) *VEGF-R2*, and *VEGF-R3* was assessed using real-time PCR. Data are presented as means ± SEM. Statistical significance was analyzed with two-tailed *t*-test (*n* = 5). **p < 0.01; ***p < 0.001.

Lymphangiogenesis is mainly induced by binding of VEGF-C and VEGF-D to VEGF-R2 and VEGF-R3^30^. We now wondered if the inhibition of CBS induced by inhibitor AOAA affects the expression of cytokines and their receptors. Therefore, HDLECs were treated with 4 mM AOAA and RNA was isolated after 24 h. We identified no significant changes in the expression of *VEGF-C* and *VEGF-D* in HDLECs after treatment 4 mM AOAA compared to control (Fig. 4a). In contrast, treatment with 4 mM AOAA results in significant downregulation of the expression of *VEGF-R2* and *VEGF-R3* in treated HDLECs compared with control cells (Fig. 4b).

In summary, these data clearly indicate that the treatment of HDLECs with the pharmacological inhibitor for CBS AOAA influences the lymphangiogenic properties of the lymphatic endothelial cells by reducing the expression of lymphangiogenic receptors VEGF-R2 and VEGF-R3. This implies, at least in vitro, a pro-lymphangiogenic role of CBS primarily via regulation of VEGF-R2 and VEGF-R3 expression on lymphatic endothelium.

### Silencing of cystathinonine β-synthase reduces proliferation, migration and tube formation in vitro

**Fig. 5:**
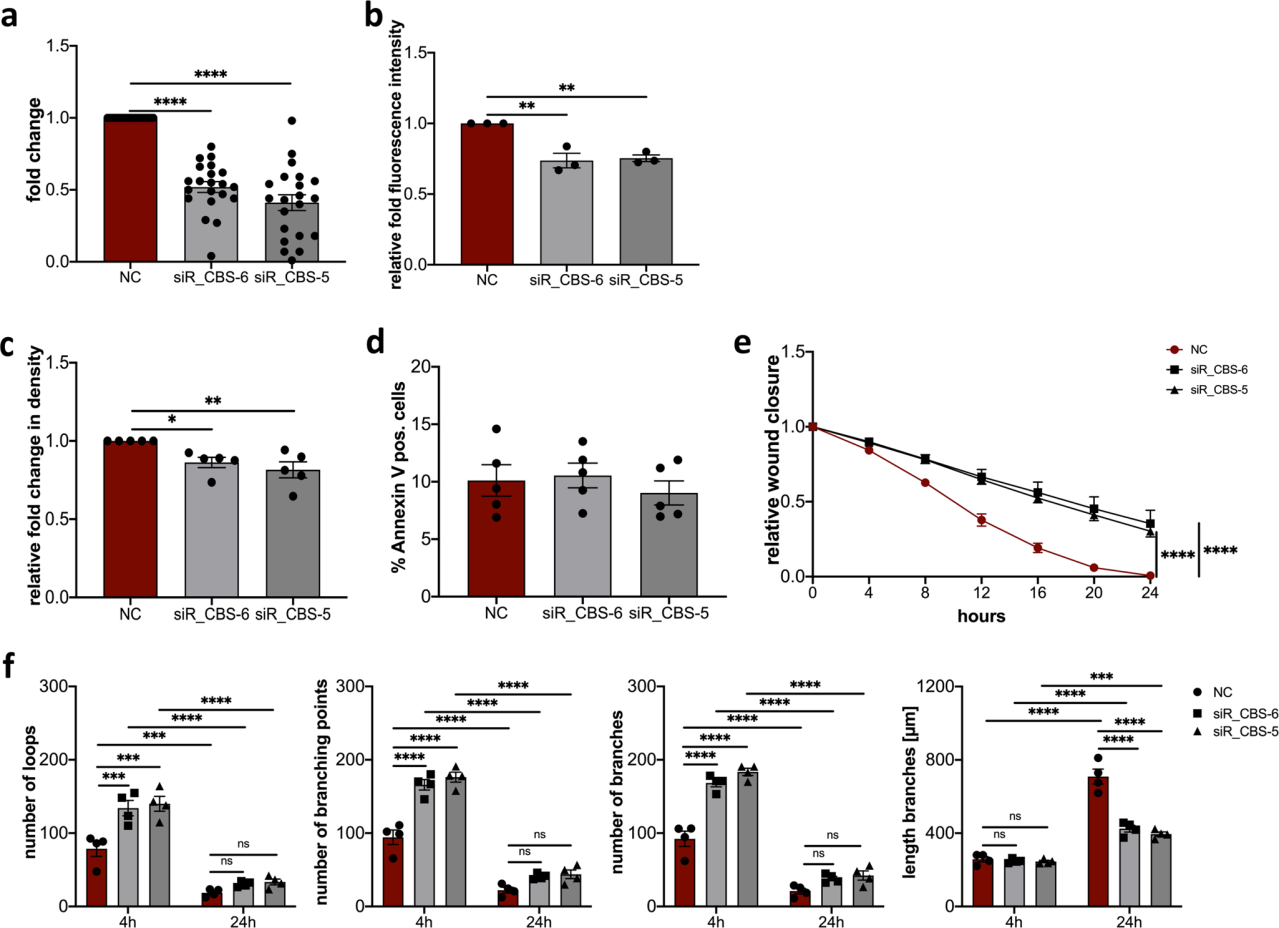
Silencing CBS affects proliferation, migration, and vessel formation of HDLECs. **a** Efficiency of CBS knockdown determined qRT-PCR 72 h post-transfection. Reduced expression of CBS in HDLECs after 72 h transfected with either siR_CBS-5, siR_CBS-6, or negative control (NC) siRNA (*n* = 21). **b** Quantification of immunofluorescence staining of HDLECs transfected with either siR_CBS-5, siR_CBS-6, or negative control (NC) siRNA stained for CBS (*n* = 3). **c** Effect of CBS silencing on HDLECs proliferation transfected with either siR_CBS-5 or siR_CBS-6 compared with NC siRNA transfected cells. Proliferation was determined 72 h after transfection by using IncuCyte Zoom (*n* = 5). **d** Quantification of flow cytometrically detected Annexin V-positive HDLECs 72 h after transfection with either siR_CBS-5, siR_CBS-6, or negative control (NC) siRNA (*n* = 5). **e** Wound healing assay with negative control (NC) siRNA, siR_CBS-5 or siR_CBS-6 siRNA treated HDLECs after 72 h transfection (*n* = 6). **f** Tube formation assay after 72 h transfection of HDLECs transfected with either negative control (NC) siRNA, siR_CBS-5 or siR_CBS-6 siRNA. Evaluation was performed after 4 h and 24 h (*n* = 4). Data are presented as means ± SEM. Statistical significance was analyzed with (**a**–**d**) one-way ANOVA and Dunnett’s multiple comparison test or (**e**, **f**) two-way ANOVA and Tukey’s multiple comparison test. *p < 0.05; **p < 0.01; ***p < 0.001; ****p < 0.0001.

The pharmacological inhibitor AOAA inhibits not only CBS but also other pyridoxal-5′-phosphate-dependent enzymes such as cystathionine-γ-lyase (CTH) also involved in the transsulfuration pathway^31,32^. To overcome this limitation, and study the direct effect of CBS we made use of specific siRNA for CBS. HDLECs transfected with either siR_CBS-5 or siR_CBS-6 result in significant downregulation of CBS expression compared to negative control (NC) siRNA 72 h after transfection (Fig. 5a).

HDLECs transfected with either siR_CBS-5 or siR_CBS-6 showed significant lower fluorescence intensity compared to negative control (NC) siRNA transfected HDLECs. This reduced fluorescence intensity is associated with reduced protein levels in the HDLECs transfected with specific siRNA (Fig. 5b). Moreover, HDLECs transfected with either siR_CBS-5 or siR_CBS-6 show significantly reduced proliferation after 24 h compared with HDLECs transfected with NC siRNA (Fig. 5c). To test whether the decreased proliferation was not caused by increased induction of apoptosis, we determined the apoptotic cells by Annexin V staining. Transfection of HDLECs with siRNA specific for CBS did not result in an increase in Annexin V-positive cells compared to negative control (NC) siRNA transfected HDLECs (Fig. 5d). Furthermore, HDLECs transfected with either siR_CBS-5 or siR_CBS-6 showed reduced p16^INK4A^ staining compared to the negative control (NC) siRNA-transfected HDLECs (Supplementary Fig. 4a, b), demonstrating that the CBS gene silencing did not induce apoptosis nor senescence in HDLECs. Now, to investigate whether the slow wound closure observed after AOAA treatment, as shown above, is indeed caused by inhibition of CBS, a scratch wound assay was performed using HDLECs specifically transfected for specific CBS siRNA. HDLECs transfected with either siR_CBS-5 or siR_CBS-6 both show significantly delayed wound closure compared to negative control (NC) siRNA transfected HDLECs (Fig. 5e).

To further define the function of CBS in HDLECs a tube formation assay was performed. Consistent with our observations in HDLECs treated with 4 mM AOAA, gene silencing of CBS with either siR_CBS-5 or siR_CBS-6 results lead to an altered formation of new tubes (Fig. 5f). CBS knock-down with either siR_CBS-5 or siR_CBS-6 resulted in a significantly increased number of loops, branching points, branches compared to NC siRNA transfected HDLECs after 4 h (Fig. 5f). In contrast, at this time point, the knockdown of CBS did not result in any change in the length of branches (Fig. 5f). The comparison of the 4 h and 24 h time points showed that here the number of loops, branching points, and branches decreases significantly over time, with branches becoming longer. After 24 h, the significant differences in the number of loops, branching points, and branches were eliminated, with now significantly shorter branches being observed after CBS knock-down compared to the HDLECs transfected with NC siRNA (Fig. 5f).

Cystathionine-γ-lyase (CTH) is the second major enzyme in the transsulfuration pathway, and is also inhibited by the pharmacological inhibitor AOAA. In order to investigate, whether CTH also had an influence on the various steps involved in lymphangiogenesis, HDLECs were transfected with specific siRNA for CTH. HDLECs transfected with either siR_CTH-8, siR_CTH-7, or siR-CTH_6 resulted in a significant down-regulation of CTH expression compared to the negative control (NC) siRNA at 72 h post-transfection (Supplementary Fig. 5a). Gene silencing of CTH in HDLECs with either siR_CTH-8, siR_CTH_7, or siR_CBS-6 had no influence on proliferation nor on wound closure at 24 h (Supplementary Fig. 5b, c).

**Fig. 6:**
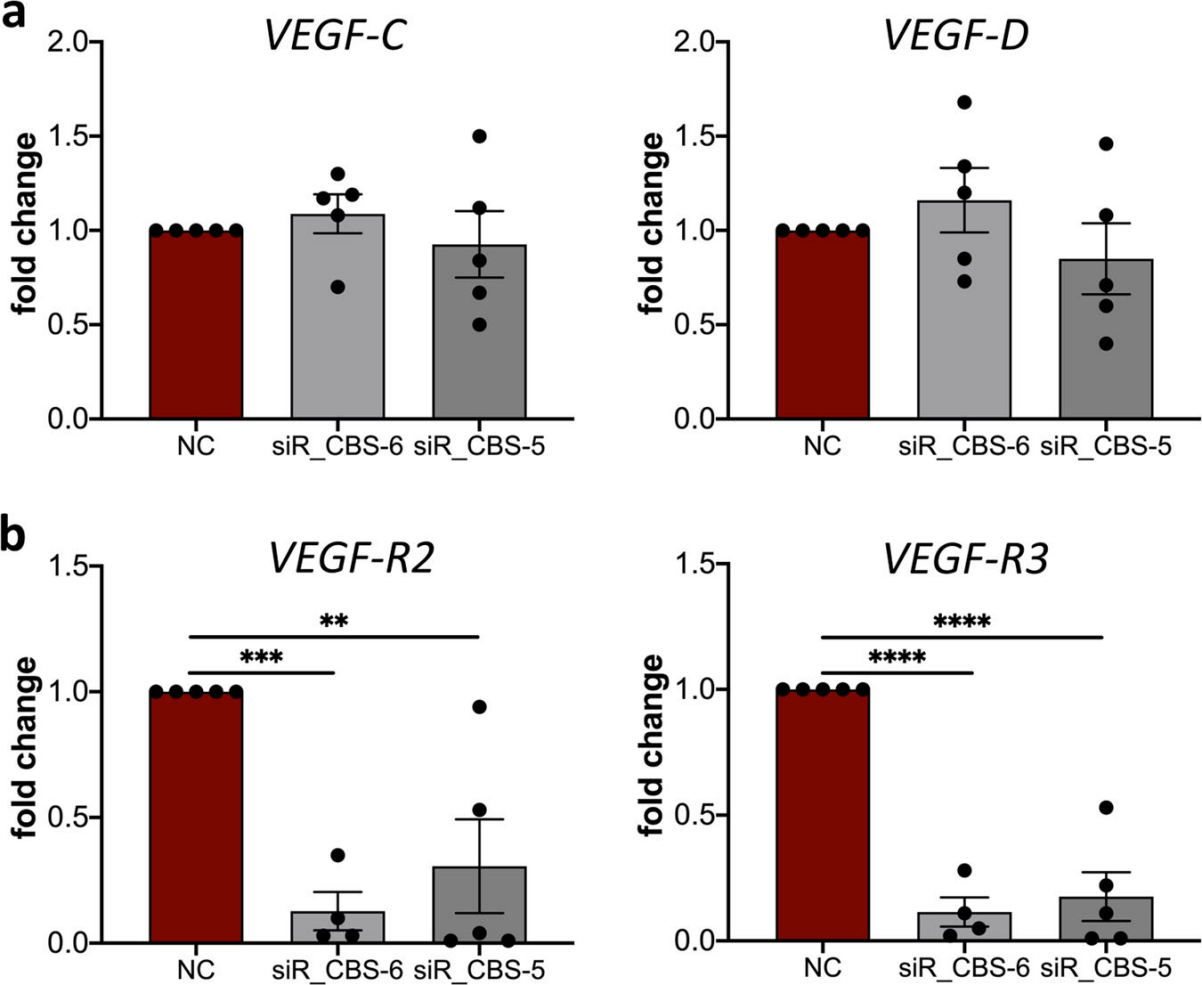
CBS knockdown reduces expression of VEGF-R2 and VEGF-R3 on HDLECs without affecting VEGF-C and VEGF-D levels. Down-regulation of CBS in HDLEC with either siR-CBS_5, siR-CBS_6, or negative control (NC). RNA was isolated 72 h after transfection and level of mRNA for (**a**) *VEGF-C*, and *VEGF-D*, (**b**) *VEGF-R2*, and *VEGF-R3* was assessed using real-time PCR. Data are presented as means ± SEM. Statistical significance was analyzed with one-way ANOVA and Dunnett’s multiple comparison test (*n* = 5). **p < 0.01; ***p < 0.001; ****p < 0.0001.

In addition to CBS and CTH, there is another important enzyme that has the ability to produce H_2_S, the 3-mercaptopyruvate sulfurtransferase (MPST). To analyze the influence of MPST on the lymphangiogenic parameters, HDLECs were transfected with specific siRNA for MPST. HDLECs transfected with either siR_MPST-7, siR_MPST-6, or siR_MPST-5, or siR_MPST-2 resulted in a significant down-regulation of MPST expression compared to the negative control (NC) siRNA at 72 h post-transfection (Supplementary Fig. 6a). Gene silencing of MPST in HDLECs with either siR_MPST-7, siR_MPST-6, or siR_MPST-5, or siR_MPST-2 had no influence on proliferation after 24 h (Supplementaty Fig. 6b).

Now, to test whether the pharmacological inhibitor AOAA acts exclusively by inhibiting CBS, gene silencing experiments were performed for either CBS, CTH, or MPST. HDLECs transfected with either siR_CBS-5 or siR_CBS-6 show significantly reduced proliferation after 24 h compared with HDLECs transfected with NC siRNA (Supplemental Fig. 7a). The treatment with 1 mM or 4 mM AOAA significantly reduces the proliferation of NC transfected HDLECs. However, no differences in proliferation were observed in HDLECs transfected with either siR_CBS-5 or siR_CBS-6 and additionally treatment with either 1 mM or 4 mM AOAA compared to the corresponding NC control (Supplementary Fig. 7b). HDLECs in which a knock down for either CTH (siR_CTH-8, siR_CTH_7, or siR_CBS-6) or MPST (siR_MPST-7, siR_MPST-6, or siR_MPST-5, or siR_MPST-2) showed corresponding downregulation of CTH or MPST compared to NC transfected HDLECs, respectively (Supplementary Fig. 7c, e). We observed here, as already shown in Supplemental Figs. 5 and 6, that transfection with the individual siRNAs had no effect on the proliferation of HDLECs. Again, additional treatment with 1 mM AOAA or 4 mM AOAA significantly reduced proliferation (Supplementary Fig. 7d, f). The data clearly show, that AOAA directly affects CBS.

To analyze whether CBS also directly affects the expression of the lymphangiogenic cytokines VEGF-C and VEGF-D and their receptors VEGF-R2 and VEGF-R3, knockdown experiments were performed in HDLECs and the expression of the cytokines and their receptors was determined by qRT-PCR (Fig. 6). The knock-down of CBS in HDLEC with either siR-CBS_5 or siR-CBS_6 both showed no influence on the expression of the lymphangiogenic cytokines *VEGF-C* and *VEGF-D* (Fig. 6a). However, after gene silencing of *CBS*, significantly decreased expression of the lymphangiogenic receptors *VEGF-R2* and *VEGF-R3* was observed on HDLECs (Fig. 6b) similar to what was observed after treatment with 4 mM AOAA.

These results indicate that CBS specifically regulates proliferation, the formation of new tubes, and migration and is involved in the expression of the lymphangiogenic cytokines receptors VEGF-R2 and VEGF-R3.

### Inhibition of CBS leads to decreased inflammation-induced lymphangiogenesis in vivo

**Fig. 7:**
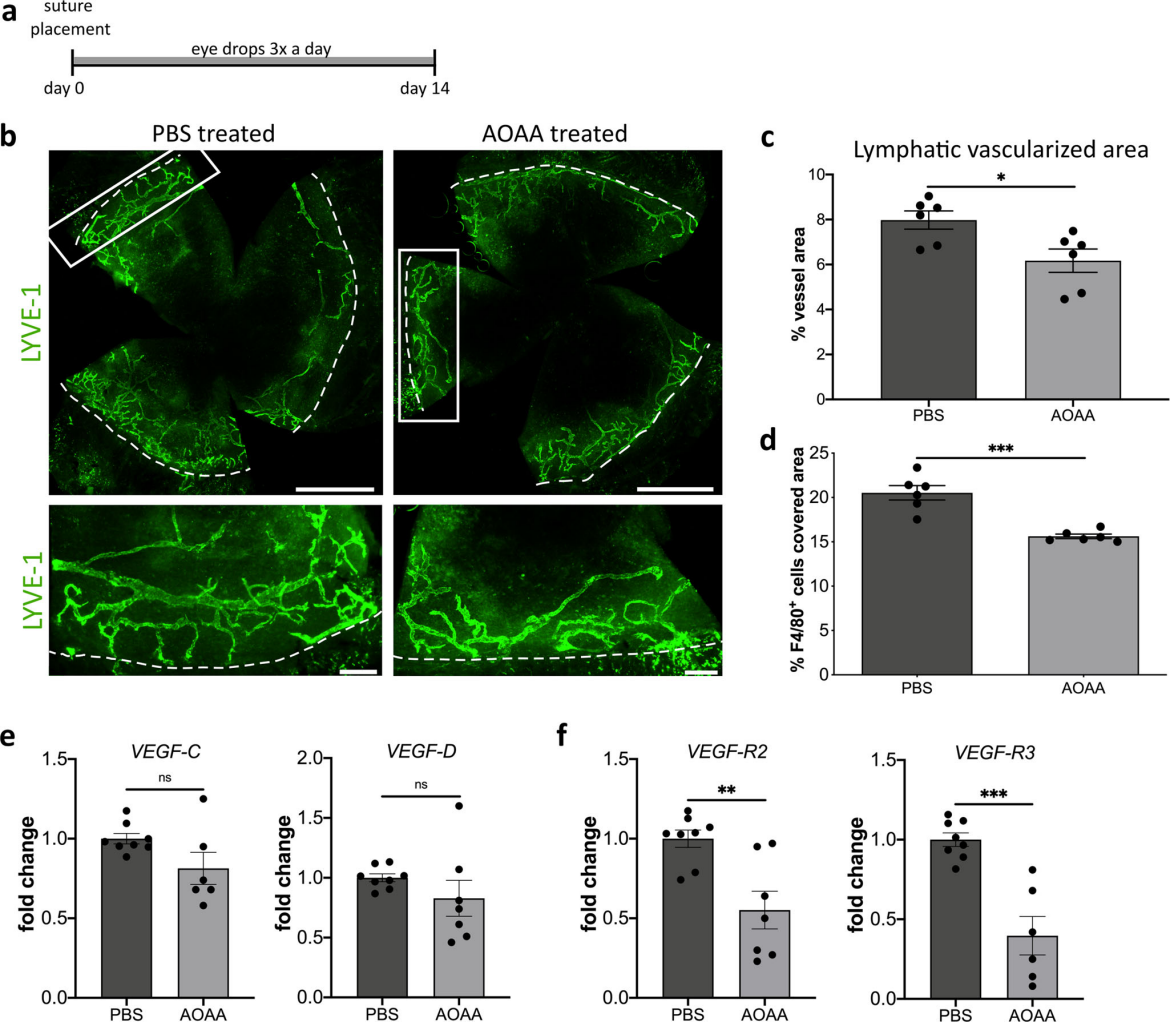
CBS-inhibition by AOAA treatment reduces inflammation-induced lymphangiogenesis in C57BL/6 N mice. **a** Schematic description of the suture‐induced model of corneal neovascularization: Intrastromal sutures were placed into the cornea of the animals (day 0). Mice were then treated with AOAA (4 mM) or PBS eyedrops 3 times a day for 2 weeks until end of experiment (day 14). **b** Representative corneal whole mounts of inflammation-induced lymphangiogenesis of C57BL/6 N stained for LYVE-1. The boxed areas in the top panels are shown in higher magnification in the bottom panels. Dashed lines show the border between the limbus and the cornea. Scale bars: 1 mm (top panel) 200 µm (lower panel). **c** Quantification of the inflammatory lymphatic vascularized area of the whole mounts. Data are expressed as means ± SEM (*n* = 6). **d** Quantification of area covered with F4/80^+^ macrophages in the inflamed cornea. Data are expressed as means ± SEM (*n* = 6). Determination of mRNA levels of (**e**) *VEGF-C*, and *VEGF-D* and (**f**) *VEGF-R2*, and *VEGF-R3* in corneas of mice treated with AOAA (4 mM) (*n* = 7) or PBS eyedrops for 14 days (*n* = 8). Data are presented as means ± SEM. Statistical significance was calculated by two-tailed *t*-test *p < 0.05; **p < 0.01; ***p < 0.001.

Since the results of inhibiting CBS by using the pharmacological inhibitor AOAA and the gene silencing were comparable, we used the pharmacological inhibitor as eye drops to investigate the influence of CBS on inflammatory lymphangiogenesis in vivo. To address this question, we used the established model of suture-induced inflammatory corneal neovascularization assay^7^ to evaluate the outgrowth of lymphatic vessels into the normally avascular cornea. For this, sutures were placed into the corneas and the animals were treated for 14 days with either AOAA (4 mM) or PBS as eye drops (Fig. 7a). To determine inflammatory lymphangiogenesis, fourteen days after the inflammatory insult corneal whole mounts were prepared and stained for the lymphatic vessel endothelial hyaluronan receptor 1 (LYVE-1). Afterward the total surface area of the vessels ingrown into the cornea of AOAA treated C57BL/6 N and PBS treated C57BL/6 N mice (Fig. 7b) was assessed. The C57BL/6 N mice treated with AOAA showed a significantly lower lymphatic surface area (Fig. 7c) as well as a significant lower area covered by F4/80^+^ macrophages (Fig. 7d) after injury compared with C57BL/6 N mice treated only with PBS, demonstrating that CBS is also involved in inflammation-induced lymphangiogenesis in vivo.

The treatment of HDLECs with AOAA reduced expression of VEGF-R2 and VEGF-R3 in vitro. This raises the question of whether the reduced inflammation-induced lymphangiogenesis caused by treatment with AOAA is also associated with a reduction in VEGF-R2 and VEGF-R3. Therefore, fourteen days after inflammatory insult, the central cornea was excited and RNA isolated for gene expression analysis. We identified no significant changes in the expression of VEGF-C and VEGF-D in the inflamed central cornea of AOAA treated C57BL/6 N compared to PBS treated C57BL/6 N mice (Fig. 7e). Consistent with the reduced corneal inflammatory lymphangiogenesis after treatment with AOAA, these corneas of AOAA-treated animals exhibit a significantly lower expression of the lymphangiogenic receptors VEGF-R2 and VEGF-R3 (Fig. 7f).

**Fig. 8:**
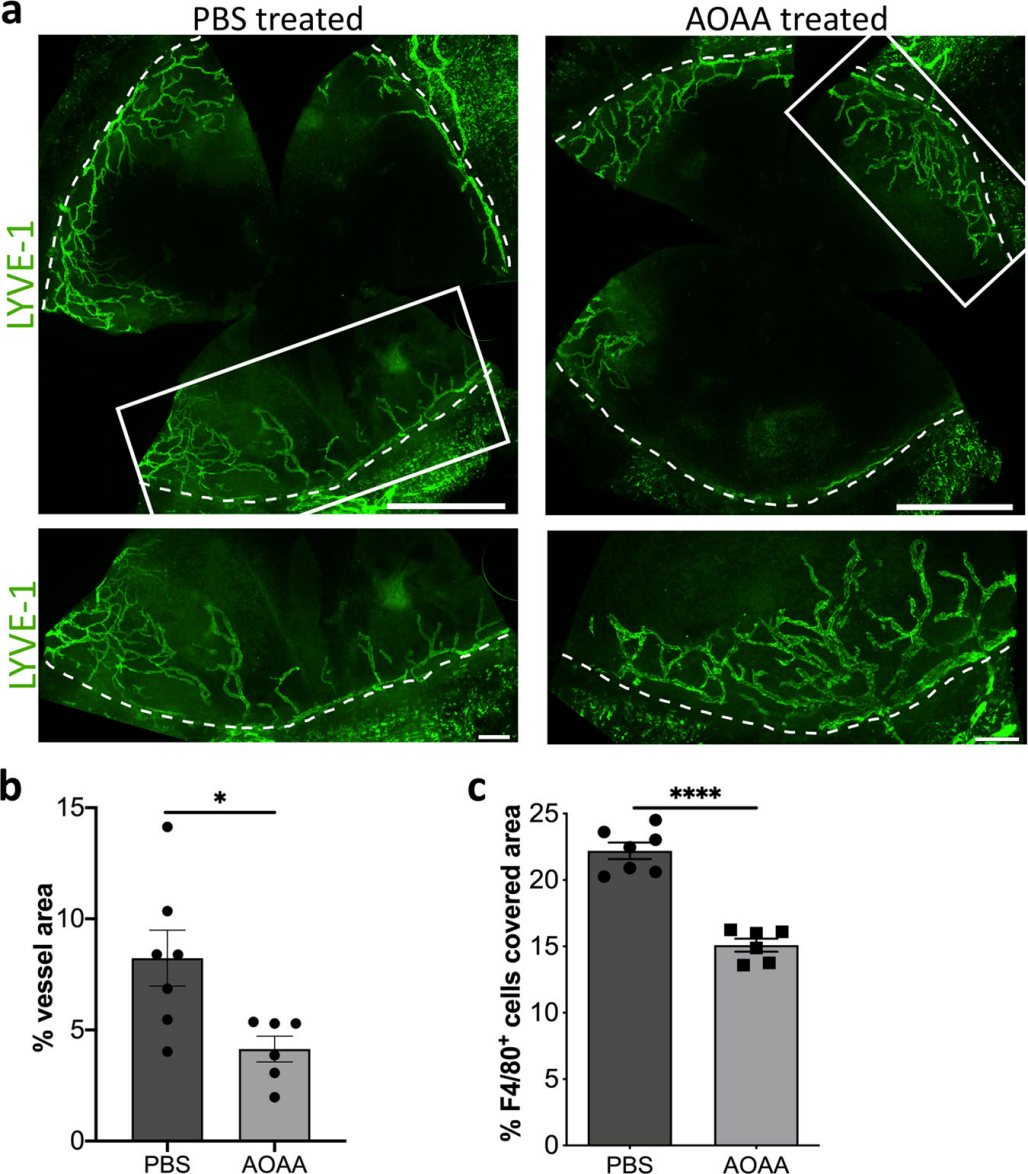
CBS-inhibition by AOAA treatment reduces inflammation-induced lymphangiogenesis in BALB/cN mice. **a** Representative corneal whole mounts of inflammation-induced lymphangiogenesis of BALB/cN stained for LYVE-1. The boxed areas in the top panels are shown in higher magnification in the bottom panels. Dashed lines show the border between the limbus and the cornea. Scale bars: 1 mm (top panel) 200 µm (lower panel). **b** Quantification of the inflammatory lymphatic vascularized area of the whole mounts. **c** Quantification of area covered with F4/80^+^ macrophages in the inflamed cornea. Data are expressed as means ± SEM (PBS *n* = 7, AOAA *n* = 6). Data are presented as means ± SEM. Statistical significance was calculated by two-tailed *t*-test *p < 0.05, ****p < 0.0001.

Since the expression of *Cbs* is 35.7-times lower in the BALB/c mice, we now wondered if the lower expression of *Cbs* in the BALB/c mice eliminates the AOAA effect. Therefore, we perform the established model of suture-induced inflammatory corneal neovascularization^7^ assay also with the BALB/c animals. Fourteen days after the inflammatory insult, the total surface area of the vessels ingrown into the cornea of AOAA treated BALB/cN and PBS treated BALB/cN mice (Fig. 8a) was assessed. Interestingly, despite the BALB/c mice showing a 35.7-times lower *Cbs* expression in the cornea the mice treated with AOAA showed a significantly lower lymphatic surface area after injury compared with BALB/c mice treated only with PBS (Fig. 8b). In addition, BALB/c mice treated with AOAA showed a significant lower area covered by F4/80^+^ macrophages compared with BALB/c mice treated only with PBS (Fig. 8c).

The result indicated that CBS has modulatory properties in inflammatory lymphangiogenesis, more specifically CBS regulated the expression of VEGF-R2 and VEGF-R3 expression in the cornea.

## Discussion

The results we present here from QTL analysis of limbal corneal lymphatic vascular morphologic features of high-lymphangiogenic C57BL/6 N and low-lymphangiogenic BALB/cN intercross and the subsequent functional in vitro and in vivo characterization allow two important conclusions. First, the QTL analysis of lymphatic vascular features between mouse strains using the corneal limbus as a model is a suitable method for the identification of novel endogenous modulators of lymphangiogenesis. Second, we could clearly demonstrate, that the identified candidate gene *cystathionine β-synthase (CBS)* exhibits pro-lymphangiogenic properties via regulation of *VEGF-R2* and *VEGF-R3*-expression. We thereby identified a novel endogenous regulator of lymphangiogenesis thus opening new treatment options for lymphangiogenesis.

In human’s cystathionine β-synthase (CBS) is a tetramer of 63 kDa subunits and responsible for the initial and rate-limiting step in the transsulfuration pathway of sulfur amino acids and also participates in the desulfuration reactions that contribute to endogenous hydrogen sulfide (H_2_S) production^33^. In addition to CBS, it has shown that two other enzymes generate H_2_S enzymatically, cystathionine-γ-lyase (CTH) and 3-mercaptopyruvate sulfurtransferase (MPST). Besides CBS, CTH also plays an important role in the second step of the transsulfuration pathway and both enzymes are pyridoxal-5 phosphate-dependent^34^. Several studies confirmed that H_2_S stimulates the triad of cellular responses responsible for endothelial cell angiogenic behaviors^34^, vascular development^35^, angiogenesis^36,37^, and epithelial-mesenchymal transition^38^. Recently, it has been shown, that H_2_S attenuates ROS and improves mitochondrial function^39^. It should be noted here, however, that CBS and CTH catalyze a number of additional reactions that do not result in H_2_S^34^.

CBS in humans is mainly expressed in the brain, liver, kidney, and pancreas^40,41^, however to a lesser extent also in other tissue, such as endocrine tissues, the gastrointestinal tract, lungs, the bladder, muscle tissues, adipose tissue, and lymphoid tissue^42^. Furthermore, expression of CBS could be detected in different compartments of the eye, like the conjunctiva, iris, ciliary body, and cornea^43^.

Primarily, CBS, under physiological conditions, is a cytosolic enzyme, although in some tissues a detectable amount of CBS can also be localized in the mitochondria^44,45^. At the cellular level, CBS is regulated by different mechanisms, for instance by transcriptional, epigenetic, post-transcriptional, or by hormonal regulation^46^. In addition, various growth and differentiation factors such as epidermal growth factor (EGF), transforming growth factor-α (TGF-α), cAMP and dexamethasone can also induce CBS expression^47^.

Cbs-deficient mice develop a severe phenotype, exhibiting growth retardation, hyperhomocysteinemia, liver steatosis, facial alopecia, loss of visceral fat, decreased bone mineralization, and early mortality^33^. Furthermore, the knock-out mice develop progressive endothelial dysfunction, such as significant vascular remodeling, increased wall thickness, and elevated blood pressure^33,48^.

In many cancer cells, metabolic and energy metabolic pathways are upregulated^49^, including CBS. This is true for breast, prostate, ovarian, liver, and colon cancer^50,51^. By altering the expression or activity of CBS, CBS has been shown to promote proliferation of colon cancer cells^50^. Furthermore, it has also been shown that CBS not only promotes tumor growth and progression, but CBS is also involved in tumor formation^52^. For this reason, many studies use pharmacological inhibitors for CBS or perform gene silencing experiments to study the function of CBS. Currently AOAA is considered the most potent pharmacological inhibitor of CBS^46^. AOAA was identified as an inhibitor of further PLP-dependent enzymes, like cystathionine-γ-lyase (CTH), alanine transaminase, glutamate decarboxylase, alanine racemase, histidine decarboxylase, D-amino acid transaminase, aspartate transaminase, and DOPA- (levodopa or l-3,4-dihydroxyphenylalanine) decarboxylase^33^. Its antitumor effects have been demonstrated using mouse xenograft models of colon^50^ and breast cancer^53^, as well as in patient-derived colon cancer xenografts^50^. Szabo et al. identified that the treatment of the colon cancer cells line HCT116 with AOAA inhibits proliferation and suppresses the migration of endothelial cells in colon cancer co-culture^50^.

Recently Saha et al. observed that the pharmacological inhibitor AOAA directly affects the proliferation of HUVECs^29^. In the current study we now use the pharmacological inhibitor AOAA to analyze the effect of CBS on lymphangiogenesis. Lymphangiogenesis is a complex process that involves different steps like proliferation, cell migration, and the organization of lymphatic endothelial cells into new capillary structures. Therefore, we treated lymphatic endothelial cells with the inhibitor and analyzed in vitro different steps involved lymphangiogenesis. The treatment of HDLECs with AOAA significantly reduces proliferation of these cells in a dose dependent manner but did not lead to induction of apoptosis. A significant reduction in proliferation of HDLEC and a pronounced delay in wound closure was observed from a concentration of 1 mM AOAA used, whereas lower concentrations had no significant effect on the proliferation of HDLECs. On the other side, the treatment of HDLECs with 1 mM AOAA did not result in altered tube formation as seen after treatment with 4 mM AOAA. Szabo et al. demonstrated decreased proliferation in colon carcinoma cells after treatment with 1 mM AOAA, while the same concentration had no effect on proliferation in non-tumorigenic colon epithelial cells^50^. In HCT116 cells, however, Hellmich et al. observed a reduced proliferation already by using a concentration of 300 µM AOAA^54^. Therefore, it appears that the inhibitory effect of AOAA is cell type-dependent.

The next steps in the lymphangiogenesis process were migration, and the organization of LECs into new capillary structures. The chosen concentration of AOAA results in delayed wound closure. However, in contrast to proliferation and migration, the inhibitor AOAA has a beneficial effect on the cellular branching behavior. These results suggested a context-dependent effect of CBS on different steps of lymphangiogenesis.

The pharmacological inhibitor AOAA does not only specifically inhibit CBS but also other pyridoxal-5′-phosphate-dependent enzymes such as cystathionine-γ-lyase (CTH)^31,32^. Endothelial cells also express CSE, which can also regulate a variety of endothelial functions^34,55^.

Therefore, gene silencing experiments were performed to analyze the effect of CBS in more detail. Lymphatic endothelial cells were transfected with siRNA specific for CBS, and 72 h after transfection, the content of Annexin V positive cells was measured and various lymphangiogenic parameters were determined. HDLECs transfected with CBS-specific siRNA showed no increase in apoptotic cells and significantly decreased proliferation, delayed wound healing, and impaired new tube formation. CBS gene silencing in HDLECs showed the same lymphangiogenic properties in vitro as lymphatic endothelial cells treated with the pharmacological inhibitor AOAA. Gene silencing experiments for the second enzyme in the transsulfurations pathway CTH displayed no involvement of this enzyme in lymphangiogenesis. Using gene silencing experiments for MPST, the third enzyme that produces H_2_S enzymatically, an involvement of MPST in lymphangiogenesis could also be ruled out.

To analyze whether the pharmacological inhibitor AOAA acts by inhibiting CBS gene silencing experiments of CBS, CTH, and MPST were performed and transfected cells were additionally treated with two different concentrations of AOAA. The obtained results clearly show, that the pharmacological inhibitor AOAA exclusively acts through inhibition of CBS.

Reduced proliferative and migratory capacity after CBS gene silencing could also be observed in HUVECs^29^, but it also induced premature senescence in these cells^56^. Zhang et al. identified that AOAA inhibits the proliferation in multiple myeloma and elevated significant the G0/G1 phase proportion of the cells^57^. Moreover, significant upregulation of the senescence-related *p21*, *Pai-1*, *Mcp1*, and *Il-6* mRNAs was observed in liver of Cbs^−/−^ mice^58^. The results obtained here show that both treatments of HDLECs with the pharmacological CBS inhibitor AOAA as well gene silencing with specific siRNA for CBS lead to a reduction in proliferation and migration and to an induction of the formation of new tubes. Furthermore, neither the treatment of HDLECs with AOAA for 24 h nor the transfection of HDLECs with specific siRNAs for CBS after 96 h leads to an induction of cellular senescence.

However, for angiogenic properties, such as proliferation, migration and tube-forming ability of endothelial cells, which are critical steps in the tissue-supporting repair and renewal of the lining of established blood vessels, growth factors of the vascular endothelial growth factor family play a very important role. The signal transduction pathway via VEGF-R2/Neuropiline (NRP)-1 activated by VEGF-A is crucial for the maintenance of endothelial cell function^59^. Lymphangiogenesis, i.e., proliferation and migration of lymphatic endothelial cells is mainly induced by binding of VEGF-C and VEGF-D to VEGF-R2 and VEGF-R3^6^. Extending our observation to the molecular level, we observed that the treatment of HDLECs either with AOAA or the transfection with specific siRNA for CBS reduces the expression of the *VEGF-R2* and *VEGF-R3* at transcriptional level. This finding can explain the reduced proliferation and migration of lymphatic endothelial cells after treatment with AOAA or transfection with specific siRNA against CBS. Saha et al. have described a role for CBS in regulating angiogenic parameters in vitro^29^, but the importance of CBS in lymphangiogenesis has not been reported before. The results obtained here show that both treatments of HDLECs with the pharmacological CBS inhibitor AOAA as well gene silencing with CBS-specific siRNA led to a reduction in proliferation and migration and induction of new tube formation, with an important role for VEGF receptor reduction.

Patients with CBS deficiency exhibit elevated homocysteine plasma level and this is commonly linked to many eye disorders, like lentia, myopia and to a lesser extent, retinal degeneration, retinal detachment, optic atrophy, glaucoma, corneal abnormalities, and cataracts^43^. Tawfik et al. observed a change in retinal vasculature in Cbs^−/−^ mice with elevated homocysteine levels and this is associated with increased VEGF mRNA and protein^60^. Persa et al. analyzed the distribution of CBS in additional different compartments in the human eye. In the eye of a 17-year-old donor, the group was able to demonstrate a strong expression of CBS in the anterior region of the eye, such as the conjunctiva, iris, ciliary body, and cornea. This distribution pattern of CBS in the anterior region of the eye remains the same during aging. In the epithelial layer of the cornea, the CBS activity is much higher than in other areas of the eye that express CBS^43^. This could be explained by environmental oxygen penetrating the corneal epithelial layer as a source of ROS generation within the cells, which can stimulate CBS activity.

To analyze the influence of CBS on inflammation-induced lymphangiogenesis, the suture-induced corneal neovascularization assay was used^7^, and inhibition of CBS was achieved by local administration of the pharmacological inhibitor AOAA as eye drop 3x daily. The placement of the suture leads to an ingrowth of lymphatic vessels into the cornea of the C57BL/6 N mice and BALB/cN mice. The reduced corneal lymphatic ingrowth of both animal strains can be attributed to the local AOAA treatment and the concomitant reduced expression of VEGF-R2 and VEGF-R3 during the suture-induced inflammatory response, since the control mice did not show this reduced lymphatic surface area after the injury.

Various in vivo and in vitro models were used to study the effect of CBS on tumor and blood vessel growth. Silencing CBS in endothelial cells leads to reduces proliferation, migration and formation of new tubes in vitro^29^ and reduces the density and the of CD31-positive blood vessel and suppresses the prevalence for larger blood vessels in a xenograft model^50^. In a xenograft model, gene silencing of CBS reduced the density of CD31-positive blood vessels and demonstrated suppressed prevalence for larger blood vessels^50^.

In this study, we identified cystathionine β-synthase as a novel endogenous regulator of lymphangiogenesis. CBS stimulates proliferation and migration in vitro, which seems to be linked to its upregulation of expression of the lymphangiogenic receptors VEGF-R2 and VEGF-R3 in lymphatic endothelial cells in vitro. CBS also increases inflammation induced lymphangiogenesis in vivo. This opens new avenues in the treatment of diseases associated with pathologic lymphangiogenesis, such as corneal graft rejection.

## Methods

### Animals

Male C57BL/6 N and female or male BALB/cN mice, aged between 8 and 10 weeks (animal facility, Center for Molecular Medicine, Cologne, Germany), were used for corneal phenotyping of the parental strains. For RNA sequencing only male mice of both strains were used. Mice used for the suture-induced inflammatory corneal neovascularization assay were 6- to 10-week-old female C57BL/6 NCrl animals (C57BL/6 N; Charles River Germany, Sulzfeld, Germany) or BALB/cAnNCrl animals (BALB/c; Charles River Germany, Sulzfeld, Germany). The experiments were approved by the local animal care committee LANUV North Rhine-Westphalia (AZ 84-02.05.2011.210 and AZ 81-02.04.2020.A199) and are in accordance with institutional and national guidelines.

### BALB/cN x C57BL/6 N Intercross

Male BALB/cN and female C57BL/6 N mice were mated to generate a heterozygous F_1_ generation. The resulting F_1_ animals were intercrossed resulting in 873 F_2_ animals (495 female, 378 male). A total of 873 corneal whole mounts from F_2_ animals between 8 and 11 weeks of age were stained for lymphatic vessels with lymphatic vessel endothelial hyaluronan receptor (LYVE)-1. A total of 795 corneas with high-quality staining (from 460 female and 335 male F_2_ animals) could be used for corneal phenotyping. For all F2 animals tail biopsies were taken and stored at −20 °C for DNA extraction with the Qiagen DNeasy Blood and Tissue Kit (Qiagen, Hilden, Germany).

Information about informative single-nucleotide polymorphism markers was retrieved from MGI (http://www.informatics.jax.org/strains_snps.shtml). QTLs were determined relative to the standard sex-averaged genetic map^61^ and translated to physical coordinates using the same data source. Using predesigned TaqMan single-nucleotide polymorphism genotyping assays (Life Technologies, Carlsbad, CA) on 384-well plates, according to the manufacturer’s instructions, all F_2_ animals were initially genotyped for 175 single-nucleotide polymorphisms evenly distributed over all recombining chromosome and distinguishing both strains. Inside the signals of the initial QTL mapping the density of markers was increased to a final number of 200 markers.

### Morphometrical analysis and quantification of the phenotype parameter vessel area, vessel length density, branching points, end points, and sprouts of parental and F_2_ generation for QTL analysis

The morphometric data used herein the publication were collected in our previous publication^22^ and reused here for deeper analysis.

### QTL mapping

Prior to QTL mapping phenotype measurements were transformed using the Box-Cox-method. For QTL mapping the R/qtl package version 1.41–6^62,63^ in the R statistics environment (https://www.R-project.org) with the Haley-Knott algorithm for the parametric method. Logarithm of the odds (LOD) thresholds for genome-wide significance were calculated based on 10.000 permutations (*α* = 0.05).

### Whole transcriptome analysis by sequencing of total RNA

Total RNA was extracted from four corneas of BALB/cN and C57BL/6N mice each using RNeasy Micro Kit (Qiagen, Hilden, Germany), according to the manufacturer ´s protocol. After quality control of the RNA samples on an Agilent 2100 Bioanalyzer (Agilent Technologies, Santa Clara, USA), 100 ng of total RNA was the input for the preparation of strand-specific total RNA sequencing libraries with the Ovation RNA-seq system for model organisms. Library preparation was performed with mouse specific reagents according to the manufacturer’s recommendations with 12 PCR cycles during library amplification, followed by library quality control again on the Bioanalyzer 2100 and additional quantification using a Qubit fluorometer (Invitrogen, Karlsruhe, Germany).

### RNA sequencing data analysis

Pooled libraries were sequenced on an Illumina Hiseq 2500 platform as 100 + 100 base paired-end reads. Raw reads were trimmed of poly-N-tails, bases with quality lower than 20 and clipped of potentially remaining sequencing adapters using cutadapt (version 1.10)^64^ and reads from (mt)-rRNA and (mt)-tRNA were filtered.

To avoid the detection of false-positive differentially expressed genes resulting from lower alignability of BALB/c sequences all SNPs and InDels from the Sanger mouse genomes project (Release 1505)^65^ for the two mouse strains were integrated into the GRCm38 reference genome using modtools (version 1.0.2)^66^. After alignment with Tophat2 (version 2.1.1)^67^ in very sensitive mode to these strain-specific pseudo-genomes the applicability of a shared high-quality standard gene model was restored by applying Lapels (version 1.1.1)^68,69^, which takes the alignment files and converts them back to the standard reference genome considering the variants initially integrated to build the pseudogenome.

Gene expression was then quantified by count on the union of exons for each gene of the Ensembl mouse gene model (Release 94) with HTSeq (version 0.7.1)^70^.

Differential expression analysis was performed as comparison of strains with DESeq2 (version 1.30)^71^ using default functionality. *P*-values were recalibrated using fdrtool (version 1.2.16)^72^ before Benjamini-Hochberg-correction for multiple testing.

### Cell culture

Adult human dermal lymphatic endothelial cells (HDLECs; Promocell, Heidelberg, Germany) were cultured in complete endothelial cell growth medium MV2 (Promocell, Heidelberg, Germany) consisting of Endothelial Cell Basal Medium MV2 containing 5% fetal calf serum (FCS), 5 ng/ml recombinant human epidermal growth factor (rh-EGF), 10 ng/ml recombinant human basic fibroblast growth factor (rh-bFGF), 20 ng/ml insulin-like growth factor, 0.5 ng/ml recombinant human vascular endothelial growth factor A 165 (rh-VEGF-A 165), 1 µg/ml ascorbic acid and 0.2 µg/ml hydrocortisone (full medium). Cells were grown in a humidified incubator (37 °C, 5% CO_2._).

HDLEC were seeded onto a 6-well plate at a cell density of 1.25 × 10^5^/well in complete endothelial cell growth medium MV2 (PromoCell, Heidelberg, Germany). On the next day, the cells were incubated in basal medium supplemented with 1% FCS (minimal medium) for 1 h. Following treatment of HDLECs with 4 mM AOAA for 24 h, cultures were washed twice with cold PBS and total RNA was extracted using the Qiagen RNeasy Total miniRNA kit (Qiagen, Hilden, Germany), according to the manufacturer’s protocol.

### Transient knock-down by siRNA transfection

HDLEC were seeded onto a 6-well plate at a cell density of 1.25 × 10^5^/well in complete endothelial cell growth medium MV2 (PromoCell, Heidelberg, Germany).

HDLECs were transfected with siRNAs targeting the CBS-coding region (Hs_CBS_5 FlexiTube siRNA (SI02777159), and Hs_CBS_6 FlexiTube siRNA (SI02777166) Qiagen, Hilden, Germany), CTH-coding region Hs_CTH_6 FlexiTube siRNA (SI04207560), Hs_CTH_7 FlexiTube siRNA (SI04235476), and Hs_CTH_8 FlexiTube siRNA (SI05460399), or MPST-coding region Hs_MPST_7 FlexiTube siRNA (SI03084571), Hs_MPST_6 FlexiTube siRNA (SI03042249), Hs_MPST_5 FlexiTube siRNA (SI03038308), and Hs_MPST_2 FlexiTube siRNA (SI00129409) at a final concentration of 75 nM using HiPerfect Transfection Reagent (Qiagen, Hilden, Germany) according to the manufacturer’s instructions. The All-Star Negative Control siRNA (Qiagen, Hilden, Germany) served as a control siRNA. After incubation for 4 h at 37 °C and 5% CO_2_ the reaction was stopped by adding 1 ml complete endothelial cell growth medium MV2 (PromoCell, Heidelberg, Germany). Proliferation, migration, and tube formation assay were performed 48 h post transfection. Transfection efficiency was determined by RT-qPCR.

### Proliferation assay

3000 normal HDLECs or HDLECs transfected with siRNA were seeded in 100 μl complete medium in 96-well plates. The plate was then left under the hood for 30 min to allow the cells to adhere. Cells were left to attach overnight. On the next day the cells were incubated in basal medium supplemented with 1% FCS (minimal medium). After 1 h, the medium was changed to either full medium or full medium containing the indicated concentrations of AOAA (Sigma Aldrich, Taufkirchen, Germany) or only full medium for siRNA transfected cells. Cell proliferation was measured as an increase of cell density by life-cell imaging every 4 h using the IncuCyte^TM^ Zoom (Essen Biosciences, Hertfordshire, UK). For statistical analysis, the fold increase in density within 24 h was calculated using the IncuCyte^TM^ software (Version 2016B and 2018A, Essen Biosciences, Hertfordshire, UK).

### Migration assay

10,000 normal HDLECs or HDLECs transfected with siRNA were seeded in 100 μl complete medium in 96-well plates. The plate was then left under the hood for 30 min to allow the cells to adhere. Cells were left to attach overnight. On the next day, the cells were incubated in basal medium supplemented with 1% FCS (minimal medium) for 1 h. Subsequently scratches were made in each well using a 10 μL pipette tip. After that, the medium was changed to either full medium or full medium containing the indicated concentrations of AOAA (Sigma Aldrich, Taufkirchen, Germany) or only full medium for siRNA transfected cells. Closing of the wound was determined by life-cell imaging every 4 h for 24 h using the IncuCyte^TM^ Zoom (Essen Biosciences, Hertfordshire, UK). The wound surface areas at each time-point were measured using ImageJ software. Wound closure of each replicate was calculated as the percentage of the healed scratch area compared to the original scratch area at 0 h.

### Tube formation assay

The tube formation assays were performed on Matrigel^®^ (Corning, Kaiserslautern, Germany) in μ-Slide angiogenesis assay (Ibidi, Martinsried, Germany) according to the manufacturer’s instructions. HDLECs were seeded at a cell density of 1 × 10^4^/well in complete endothelial cell growth medium MV2 (Promocell, Heidelberg, Germany) containing the indicated concentrations of AOAA (Sigma Aldrich, Taufkirchen, Germany). For determination of tube formation of transfected HDLECs the cells were seeded at a cell density of 1 × 10^4^/well in complete endothelial cell growth medium MV2 (Promocell, Heidelberg, Germany). Images were taken after 4 h and 24 h using a Zeiss Primo Vert inverted microscope fitted with an AxioCam ERc5s camera. The number of branches, loops, and branching points and the length of the branches were determined and quantified manually.

### Analysis of mRNA expression by Real Time Quantitative PCR

For mRNA analysis, complementary DNA (cDNA) syntheses were performed with RevertAid First-Strand Synthesis cDNA Synthese Kit (ThermoScientific, Langenselbold, Germany). Real-time Polymerase Chain Reaction (RT-PCR) was performed in triplets in a total volume of 20 μl, on a BioRad CFX96 (BioRad, Munich, Germany) using the quantitative (q)PCR SsoFast EvaGreen Supermix kit (BioRad, Munich, Germany) and specific primers for: β*2-macroglobulin:* forward 5’-AGG ACT GGT CTT TCT ATC TCT TG-3’, reverse 5’-CGG CAT CTT CAA ACC TCC AT-3’; VEGF-C: forward 5’-GCC TGT GAA TGT ACA GAA AGT CC-3’, reverse 5’-AAT ATG AAG GGA CAC AAC GAC AC-3’; VEGF-D: forward 5’-CCG CCA TCC ATA CTC AAT TAT C-3’, reverse 5’-CCA TAG CAT GTC AAT AGG ACA GAG -3’; VEGF-R2: forward 5’-GCG ATG GCC TCT TCT GTA AG-3’, reverse 5’-ACA CGA CTC CAT GTT GGT CA-3’; VEGF-R3: forward 5’-GGT ACA TGC CAA CGA CAC AG-3’, reverse 5’-CTC AAA GTC TCT CAC GAA CAC G-3’, CBS: forward 5’-TCA TCG TGA TGC CAG AGA AG-3’, reverse 5’-TTG GGG ATT TCG TTC TTC AG-3’. β*2-macroglobulin* was used as endogenous control for normalization. Expression levels of the target genes are displayed as values relative to the levels found in unstimulated cells.

### Apoptosis assay

For the apoptosis assays the BioLegend FITC Annexin V apoptosis detection kit with 7-aminoactinomycin D (7-AAD) was used according to the instructions of the manufacturer. HDLEC were seeded onto a 6-well plate at a cell density of 1.25 × 10^5^/well in complete endothelial cell growth medium MV2 (PromoCell, Heidelberg, Germany). On the next day, the cells were incubated in basal medium supplemented with 1% FCS (minimal medium) for 1 h. Following treatment of HDLECs with different concentrations of AOAA (0.25 mM, 0.5 mM, 1 mM, 2 mM, 4 mM) for 24 h, cells were harvested, stained and cell samples were analyzed on a Bioscience FACSCanto II Flow Cytometer (BD Biosciences, Heidelberg, Germany). Data were analyzed using the FlowJo 8.7.3 software (Beckton Dickenson, Ashland, USA).

### Immunofluorescence staining

30,000 normal HDLECs or HDLECs transfected with siRNA were seeded in 500 μl complete medium on coverslips in a 24-well plate. Cells were left to attach overnight. On the next day the cells were incubated in basal medium supplemented with 1% FCS (minimal medium). After one hour, the medium was changed to either full medium or full medium containing the indicated concentrations of AOAA (Sigma Aldrich, Taufkirchen, Germany) or only full medium for siRNA transfected cells for another 24 h.

For immunofluorescence staining, HDLECs were washed twice with phosphate-buffered saline (PBS) containing 1 mM MgCl_2_ and 0.1 mM CaCl_2_ (PBS^++^). Subsequently, cells were fixed with 4% paraformaldehyde for 20 min, permeabilized with PBS^++^ containing 0.1% Triton X-100 (PBS^T++^) for 5 min, quenched with 50 mM NH_4_Cl in PBS^T++^ and blocked with PBS^T++^ containing 1% BSA (VWR, Langenfeld, Germany) for 1 h. Immunostaining was performed using the CBS (Santa Cruz, Heidelberg, Germany) and p16 (Abcam, Amsterdam, Netherlands) antibody. Cells were incubated for 45 min with the specific first antibody raised against the protein of interest and then with the secondary antibody conjugated with goat anti mouse Alexa Fluor 488 (Invitrogen, Darmstadt, Germany) and goat anti rabbit-Cy3 (Dianova, Hamburg, Germany). Cells were mounted with DAKO fluorescent mounting medium (DAKO). All stainings were performed at room temperature and stored at 4 °C in the dark. Images were assembled automatically with a fluorescence microscope (Olympus BX63). Quantification was performed MATLAB R2021a (MathWorks Inc., Natick, USA).

### Evaluation of fluorescence intensity

Images of the fluorescent cells were loaded into MATLAB software (The MathWorks, Inc., Natick, MA) for the determination of fluorescence intensity. First, the background signal in the image was subtracted using a top-hat algorithm. Then, the image was converted to a binary image using a threshold based on the saturation intensity value. Contiguous cells were separated using the watershed algorithm, and the mean fluorescence intensity was calculated. DAPI was used to determine the total number of cells in each image.

### Suture-Induced Inflammatory Corneal Neovascularization Assay

The mouse model of suture-induced inflammatory corneal neovascularization was used, as described previously^73^ with modifications. Briefly, before corneal surgery, each animal was deeply anesthetized by intraperitoneal injection of Ketamine (Ketanest-S) [100 mg/kg bodyweight] and Xylazine (Rompun) [10 mg/kg bodyweight] (injection volume max. 0.1 ml/10 g KG). Subsequently three 11-0 nylon sutures (Serag Wiessner, Naila, Germany) were placed intrastromally, with two stromal incursions extending over 120° of corneal circumference each. The outer point of suture placement was chosen near the limbus, and the inner suture point was chosen near the corneal center equidistant from the limbus to obtain standardized vascularization responses. To compare the lymphangiogenic responses, the sutures were left in place for 14 days. During this time animals were treated with eye drops containing either AOAA (4 mM) or PBS three times a day. Mice were sacrificed and corneas were histologically analyzed on day 14.

### Preparation of Corneal Whole Mounts and Immunohistochemistry

Excised corneas were rinsed in phosphate-buffered saline solution (PBS) and fixed 4% PFA (Alfa Aesar, Kandel, Germany) 1 h at room temperature. The whole mounts were washed trice with phosphate buffered saline (PBS) containing 1 mM MgCl_2_, 0.1 M CaCl_2_ and 0.1% Triton X-100 (PBS^++T^). Blocking for non-specific binding-sites was performed for 2 h at room temperature with 2% Bovine Serum Albumin (BSA) in PBS^++T^ on the shaker. After that the corneas were stained overnight at 4 °C with a rabbit anti-mouse LYVE-1 antibody (1:300 dilution; AngioBio Co., Del Mar, CA) and CD31 (1:200 dilution; OriGene Technologies GmbH, Herford, Germany) in 2% BSA in PBS^++T^. The next day, corneas were rinsed trice in PBS^++T^ and LYVE-1 was detected with a secondary antibody goat anti-rabbit AlexaFluor 488 (1:500 dilution; Invitrogen, Darmstadt, Germany) and CD31 was detected with goat anti-rat AlexaFluor 555 (1:500 dilution; Invitrogen, Darmstadt, Germany). After that the corneas were stained overnight at 4 °C with F4/80-AlexaFluor647 (1:100 dilution; BioLegend, Koblenz, Germany). Whole mounts were subsequently washed three times with PBS^++T^ and after a final washing step with PBS, corneal whole mounts were transferred to SuperFrost slides (Menzel-Glaser, Braunschweig, Germany), covered with Dako fluorescent mounting medium (Dako, Hamburg, Germany), and stored at 4 °C in the dark. Images were assembled automatically with a fluorescence microscope (Olympus BX63; Olympus Deutschland GmbH).

### Determination of Inflammatory Limbal Lymphatic Vessel Area

The surface area from whole mounts with inflammation-induced neovascularization were determined form treated and untreated mice. The area covered with lymphatic vessels was detected with an algorithm established in the image analyzing program cell^F 3.4: before analysis, gray value images of the whole-mount images were modified by several filters^27^. Lymphatic vessels were detected by a threshold setting, including the bright vessels and excluding the dark background, as described previously^27^.

Sprouts refer to extensions from the physiologically lymphatic-vascularized conjunctiva into the physiologically alymphatic cornea, with the limbus being the border. End points were defined as final points (extremities) of a lymph vessel structure independent of the orientation of the lymph vessel within the vessel network. The number of branching points, end points, and sprouts was counted manually using cellF^3.4 and related to the total corneal area (branching points, end points, and sprouts/mm^2^).

### RNA isolation from corneas and reverse transcription polymerase chain reaction

Total RNA isolated from the cornea was performed by using the RNA-easy micro kit (Qiagen, Hilden, Germany). Only central corneas were analyzed, as the samples were collected by excising the cornea above the limbus. Traces of genomic DNA were removed by DNase digestion with the RNase-free DNase Set (Qiagen, Hilden Germany). Subsequently, RNA was reverse-transcribed into single-stranded cDNA using the First Strand cDNA Synthesis Kit (ThermoScientific, Langenselbold, Germany) according to manufacturers’ protocol. Real-time PCR was performed using PowerTrack SYBR Green Master Mix (ThermoScientific, Langenselbold, Germany) and Applied Bioscience Quantstudio 6 (ThermoScientific, Langenselbold, Germany), and specific murine primers for *Rsp29:* forward 5’-GAG CAG ACG CGG CAA-3’, reverse 5’-CCT TTC TCC TCG TTG GG C-3’; *Vegf-a:* forward 5´- CAT GGA TGT CTA CCA GCG AAG -3´, reverse: 5´- CAT GGT GAT GTT GCT CTC TGA C -3´; Vegf-c: forward: 5´- AGA ACG TGT CCA AGA AAT CAG C -3´, reverse: 5´-ATG TGG CCT TTT CCA ATA CG-3´; Vegf-d forward: 5´-ATG GCG GCT AGG TGA TTC C-3´, reverse: 5´-CCC TTC CTT TCT GAG TGC TG-3´; *Vegf-r2:* forward 5´-ATT CTG GAC TCT CCC TGC CTA C-3´, reverse: 5´-GCT CTT TCG CTT ACT GTT CTG G-3´; *Vegf-r3*: forward 5´-GTC CCT CTA CTT CCA ACT GCT TC-3´, reverse: 5´-CAC TCC TCC TCT GTG ACT TTG AG-3´ were used. Rsp29 were used as endogenous control for normalization.

### Statistics and reproducibility

Statistical analysis of the functional studies was performed with GraphPad Prism software versions 8 (GraphPad Software, San Diego, CA). All the functional in vitro experiments were performed at least in triplicate with at least four technical replicates within each experiment. For animal experiments, sample size was set to 5 individual animals. Data are presented as the mean ± standard error of the mean (SEM) (see figure legends for additional information). The figure legends give full information about the number of independent biological replicates (*n*) analyzed and the type of statistical test, which was used to calculate significance. Statistical significance was analyzed with the two-tailed *t*-test, with the one-way analysis of variance and Dunnett multiple comparison test or with two-way ANOVA and Tukey’s multiple comparison test (see figure legends for additional information). Statistical significance is presented as *p < 0.05, **p < 0.01, ***p < 0.001, ****p < 0.0001.

### Reporting summary

Further information on research design is available in the Nature Research Reporting Summary linked to this article.

## Supplementary information

Supplementary Information

Description of Additional Supplementary Files

Supplementary Data 1

Supplementary Data 2

Reporting Summary

## Data Availability

The source data for all graphs and charts are provided in the Supplementary Data 2. Sequencing data and normalized expression values were uploaded to the Gene Expression Omnibus at GSE178730^74^.

## References

[1] Oliver G, Kipnis J, Randolph GJ, Harvey NL (2020). The lymphatic vasculature in the 21(st) century: novel functional roles in homeostasis and disease. Cell.

[2] Cursiefen C (2002). Lymphatic vessels in vascularized human corneas: immunohistochemical investigation using LYVE-1 and podoplanin. Invest Ophthalmol. Vis. Sci..

[3] Cursiefen C (2006). Nonvascular VEGF receptor 3 expression by corneal epithelium maintains avascularity and vision. Proc. Natl Acad. Sci. USA.

[4] Scavelli C, Vacca A, Di Pietro G, Dammacco F, Ribatti D (2004). Crosstalk between angiogenesis and lymphangiogenesis in tumor progression. Leukemia.

[5] Simons M, Gordon E, Claesson-Welsh L (2016). Mechanisms and regulation of endothelial VEGF receptor signalling. Nat. Rev. Mol. Cell Biol..

[6] Clahsen, T., Buttner, C., Hatami, N., Reis, A. & Cursiefen, C. Role of endogenous regulators of hem- and lymphangiogenesis in corneal transplantation. *J. Clin. Med.* **9**, 10.3390/jcm9020479 (2020). 10.3390/jcm9020479 PMC7073692 32050484

[7] Cursiefen C (2004). VEGF-A stimulates lymphangiogenesis and hemangiogenesis in inflammatory neovascularization via macrophage recruitment. J. Clin. Invest.

[8] Tammela T, Alitalo K (2010). Lymphangiogenesis: Molecular mechanisms and future promise. Cell.

[9] Oka M (2008). Inhibition of endogenous TGF-beta signaling enhances lymphangiogenesis. Blood.

[10] Zampell JC (2012). Lymphatic function is regulated by a coordinated expression of lymphangiogenic and anti-lymphangiogenic cytokines. Am. J. Physiol. Cell Physiol..

[11] Albuquerque RJ (2009). Alternatively spliced vascular endothelial growth factor receptor-2 is an essential endogenous inhibitor of lymphatic vessel growth. Nat. Med..

[12] Singh N (2013). Soluble vascular endothelial growth factor receptor 3 is essential for corneal alymphaticity. Blood.

[13] Makinen T (2001). Inhibition of lymphangiogenesis with resulting lymphedema in transgenic mice expressing soluble VEGF receptor-3. Nat. Med..

[14] Cursiefen C (2011). Thrombospondin 1 inhibits inflammatory lymphangiogenesis by CD36 ligation on monocytes. J. Exp. Med..

[15] Cursiefen C (2004). Roles of thrombospondin-1 and -2 in regulating corneal and iris angiogenesis. Invest Ophthalmol. Vis. Sci..

[16] Heishi T (2010). Endogenous angiogenesis inhibitor vasohibin1 exhibits broad-spectrum antilymphangiogenic activity and suppresses lymph node metastasis. Am. J. Pathol..

[17] Watanabe K (2004). Vasohibin as an endothelium-derived negative feedback regulator of angiogenesis. J. Clin. Invest.

[18] Wong HL (2016). MT1-MMP sheds LYVE-1 on lymphatic endothelial cells and suppresses VEGF-C production to inhibit lymphangiogenesis. Nat. Commun..

[19] Xu Y (2010). Neuropilin-2 mediates VEGF-C-induced lymphatic sprouting together with VEGFR3. J. Cell Biol..

[20] Reuer T (2018). Semaphorin 3F modulates corneal lymphangiogenesis and promotes corneal graft survival. Invest Ophthalmol. Vis. Sci..

[21] Zhuo W (2010). Endostatin inhibits tumour lymphangiogenesis and lymphatic metastasis via cell surface nucleolin on lymphangiogenic endothelial cells. J. Pathol..

[22] Buttner C (2019). Tyrosinase is a novel endogenous regulator of developmental and inflammatory lymphangiogenesis. Am. J. Pathol..

[23] Regenfuss B (2015). The naive murine cornea as a model system to identify novel endogenous regulators of lymphangiogenesis: TRAIL and rtPA. Lymphat Res Biol..

[24] Hos D, Schlereth SL, Bock F, Heindl LM, Cursiefen C (2015). Antilymphangiogenic therapy to promote transplant survival and to reduce cancer metastasis: what can we learn from the eye. Semin Cell Dev. Biol..

[25] Cursiefen C, Chen L, Dana MR, Streilein JW (2003). Corneal lymphangiogenesis: evidence, mechanisms, and implications for corneal transplant immunology. Cornea.

[26] Regenfuss B (2010). Genetic heterogeneity of lymphangiogenesis in different mouse strains. Am. J. Pathol..

[27] Bock F (2008). Improved semiautomatic method for morphometry of angiogenesis and lymphangiogenesis in corneal flatmounts. Exp. Eye Res.

[28] Gaudet P, Livstone MS, Lewis SE, Thomas PD (2011). Phylogenetic-based propagation of functional annotations within the Gene Ontology consortium. Brief. Bioinform.

[29] Saha S (2016). Cystathionine beta-synthase regulates endothelial function via protein S-sulfhydration. FASEB J.: Off. Publ. Federation Am. Societies Exp. Biol..

[30] Otrock ZK, Makarem JA, Shamseddine AI (2007). Vascular endothelial growth factor family of ligands and receptors: review. Blood Cells Mol. Dis..

[31] Asimakopoulou A (2013). Selectivity of commonly used pharmacological inhibitors for cystathionine beta synthase (CBS) and cystathionine gamma lyase (CSE). Br. J. Pharm..

[32] Stottrup NB (2010). Inhibition of the malate-aspartate shuttle by pre-ischaemic aminooxyacetate loading of the heart induces cardioprotection. Cardiovasc Res..

[33] Zuhra, K., Augsburger, F., Majtan, T. & Szabo, C. Cystathionine-beta-synthase: molecular regulation and pharmacological inhibition. *Biomolecules* **10**, 10.3390/biom10050697 (2020). 10.3390/biom10050697 PMC7277093 32365821

[34] Kanagy NL, Szabo C, Papapetropoulos A (2017). Vascular biology of hydrogen sulfide. Am. J. Physiol. Cell Physiol..

[35] Jiang W (2021). H2S promotes developmental brain angiogenesis via the NOS/NO pathway in zebrafish. Stroke Vasc. Neurol..

[36] Majumder A (2018). Hydrogen sulfide improves postischemic neoangiogenesis in the hind limb of cystathionine-beta-synthase mutant mice via PPAR-gamma/VEGF axis. Physiol. Rep..

[37] Qi, Q. R. et al. Enhanced stromal cell CBS-H2S production promotes estrogen-stimulated human endometrial angiogenesis. *Endocrinology* **161**, 10.1210/endocr/bqaa176 (2020). 10.1210/endocr/bqaa176 PMC7575054 32987401

[38] Wang M, Yan J, Cao X, Hua P, Li Z (2020). Hydrogen sulfide modulates epithelial-mesenchymal transition and angiogenesis in non-small cell lung cancer via HIF-1alpha activation. Biochem Pharm..

[39] Rao G (2020). Cystathionine beta synthase regulates mitochondrial dynamics and function in endothelial cells. FASEB J.: Off. Publ. Federation Am. Societies Exp. Biol..

[40] Bao L, Vlcek C, Paces V, Kraus JP (1998). Identification and tissue distribution of human cystathionine beta-synthase mRNA isoforms. Arch. Biochem Biophys..

[41] Kabil O, Vitvitsky V, Xie P, Banerjee R (2011). The quantitative significance of the transsulfuration enzymes for H2S production in murine tissues. Antioxid. redox Signal..

[42] Uhlen M (2015). Proteomics. Tissue-based map of the human proteome. Science.

[43] Persa C, Osmotherly K, Chao-Wei Chen K, Moon S, Lou MF (2006). The distribution of cystathionine beta-synthase (CBS) in the eye: implication of the presence of a trans-sulfuration pathway for oxidative stress defense. Exp. Eye Res.

[44] Szabo C (2014). Regulation of mitochondrial bioenergetic function by hydrogen sulfide. Part I. Biochemical and physiological mechanisms. Br. J. Pharm..

[45] Teng H (2013). Oxygen-sensitive mitochondrial accumulation of cystathionine beta-synthase mediated by Lon protease. Proc. Natl Acad. Sci. USA.

[46] Hellmich MR, Coletta C, Chao C, Szabo C (2015). The therapeutic potential of cystathionine beta-synthetase/hydrogen sulfide inhibition in cancer. Antioxid. redox Signal..

[47] Enokido Y (2005). Cystathionine beta-synthase, a key enzyme for homocysteine metabolism, is preferentially expressed in the radial glia/astrocyte lineage of developing mouse CNS. FASEB J.: Off. Publ. Federation Am. Societies Exp. Biol..

[48] Eberhardt RT (2000). Endothelial dysfunction in a murine model of mild hyperhomocyst(e)inemia. J. Clin. Invest.

[49] Martinez-Outschoorn UE, Peiris-Pages M, Pestell RG, Sotgia F, Lisanti MP (2017). Cancer metabolism: a therapeutic perspective. Nat. Rev. Clin. Oncol..

[50] Szabo C (2013). Tumor-derived hydrogen sulfide, produced by cystathionine-beta-synthase, stimulates bioenergetics, cell proliferation, and angiogenesis in colon cancer. Proc. Natl Acad. Sci. USA.

[51] Zhu H, Blake S, Chan KT, Pearson RB, Kang J (2018). Cystathionine beta-synthase in physiology and cancer. BioMed. Res. Int..

[52] Phillips CM (2017). Upregulation of cystathionine-beta-synthase in colonic epithelia reprograms metabolism and promotes carcinogenesis. Cancer Res.

[53] Thornburg JM (2008). Targeting aspartate aminotransferase in breast cancer. Breast Cancer Res..

[54] Hellmich, M. R. et al. Efficacy of novel aminooxyacetic acid prodrugs in colon cancer models: towards clinical translation of the cystathionine beta-synthase inhibition concept. *Biomolecules* **11**, 1–17 (2021). 10.3390/biom11081073 PMC8394431 34439739

[55] Szabo C (2017). Hydrogen sulfide, an enhancer of vascular nitric oxide signaling: mechanisms and implications. Am. J. Physiol. Cell Physiol..

[56] Albertini E, Koziel R, Durr A, Neuhaus M, Jansen-Durr P (2012). Cystathionine beta synthase modulates senescence of human endothelial cells. Aging (Albany NY).

[57] Zhang M (2020). Cystathionine beta synthase/hydrogen sulfide signaling in multiple myeloma regulates cell proliferation and apoptosis. J. Environ. Pathol. Toxicol. Oncol..

[58] Utyro, O., Perla-Kajan, J. & Jakubowski, H. The Cbs locus affects the expression of senescence markers and mtDNA copy number, but not telomere dynamics in mice. *Int. J. Mol. Sci.* **21**10.3390/ijms21072520 (2020). 10.3390/ijms21072520 PMC7177707 32260476

[59] Soker S, Miao HQ, Nomi M, Takashima S, Klagsbrun M (2002). VEGF165 mediates formation of complexes containing VEGFR-2 and neuropilin-1 that enhance VEGF165-receptor binding. J. Cell Biochem.

[60] Tawfik A (2013). Alterations of retinal vasculature in cystathionine-Beta-synthase mutant mice, a model of hyperhomocysteinemia. Invest Ophthalmol. Vis. Sci..

[61] Cox A (2009). A new standard genetic map for the laboratory mouse. Genetics.

[62] Arends D, Prins P, Jansen RC, Broman KW (2010). R/qtl: high-throughput multiple QTL mapping. Bioinformatics.

[63] Broman KW, Wu H, Sen S, Churchill GA (2003). R/qtl: QTL mapping in experimental crosses. Bioinformatics.

[64] Chen C, Khaleel SS, Huang H, Wu CH (2014). Software for pre-processing Illumina next-generation sequencing short read sequences. Source Code Biol. Med..

[65] Keane TM (2011). Mouse genomic variation and its effect on phenotypes and gene regulation. Nature.

[66] Huang, S., Kao, C.-Y., McMillan, L. & Wang, W. In Proceedings of the International Conference on Bioinformatics, Computational Biology and Biomedical Informatics 595–604 (Association for Computing Machinery, Wshington DC, USA, 2013).

[67] Kim D (2013). TopHat2: accurate alignment of transcriptomes in the presence of insertions, deletions and gene fusions. Genome Biol..

[68] Holt, J., Huang, S., McMillan, L. & Wang, W. In Proceedings of the International Conference on Bioinformatics, Computational B*iology and Biomedical Informatics* 605–612 (Association for Computing Machinery, Wshington DC, USA, 2013).

[69] Huang, S., Holt, J., Kao, C. Y., McMillan, L. & Wang, W. A novel multi-alignment pipeline for high-throughput sequencing data. *Database (Oxford*) 2014, 10.1093/database/bau057 (2014). 10.1093/database/bau057 PMC4062837 24948510

[70] Anders S, Pyl PT, Huber W (2015). HTSeq–a Python framework to work with high-throughput sequencing data. Bioinformatics.

[71] Love MI, Huber W, Anders S (2014). Moderated estimation of fold change and dispersion for RNA-seq data with DESeq2. Genome Biol..

[72] Strimmer K (2008). fdrtool: a versatile R package for estimating local and tail area-based false discovery rates. Bioinformatics.

[73] Bock F (2007). Bevacizumab as a potent inhibitor of inflammatory corneal angiogenesis and lymphangiogenesis. Invest Ophthalmol. Vis. Sci..

[74] Hatamie, N. et al. Cystathionine β-synthase as novel endogenous regulator of lymphangiogenesis via modulating VEGF receptor 2 and 3. https://www.ncbi.nlm.nih.gov/geo/query/acc.cgi?acc=GSE178730. 10.1038/s42003-022-03923-7 PMC9464209 36088423

