# Supplementary material for: Cystathionine β-synthase as novel endogenous regulator of lymphangiogenesis via modulating VEGF receptor 2 and 3
Source: Commun Biol. 2022 Sep 10;5:950. doi: 10.1038/s42003-022-03923-7 (PMC9464209; doi:10.1038/s42003-022-03923-7)
Supplement: Supplementary file 1 — Supplementary Information [file 42003_2022_3923_MOESM1_ESM.pdf]

Supplementary Figures and Tables:

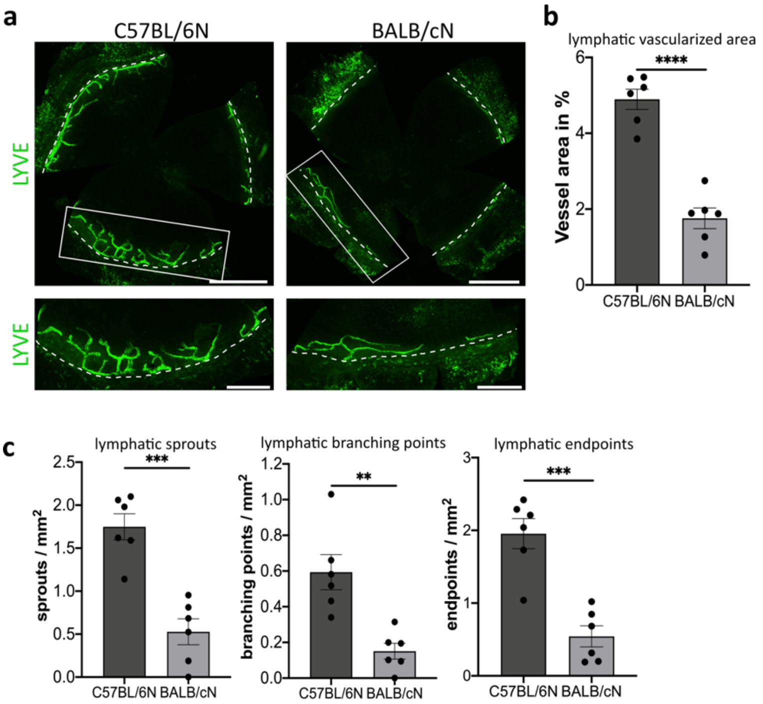

Supplementary Figure 1: Resting limbal lymphatic vasculature in parental strains of low-lymphangiogenic BALB/cN and high-lymphangiogenic C57BL/6N Representative whole mounts of the murine cornea from **(A)** C57BL/6N and BALB/cN stained for LYVE-1. The boxed areas in the top panels are shown in higher magnification in the bottom panels. Dashed lines show the border between the limbus and the cornea. Scale bars: 1 mm top panels and 500 µm bottom panels) **(B)** Quantification of the lymphatic vascularized area of the whole mounts. **(C)** Further characterization of the lymphatic vessel architecture by determination of the number of sprouts, the number of branching points, and the number of end points. Data are expressed as means ± SEM (n=6). Data are presented as means ± SEM. Statistical significance was calculated by two-tailed t-test **p<0.01, ***p<0.001, ****p<0.001

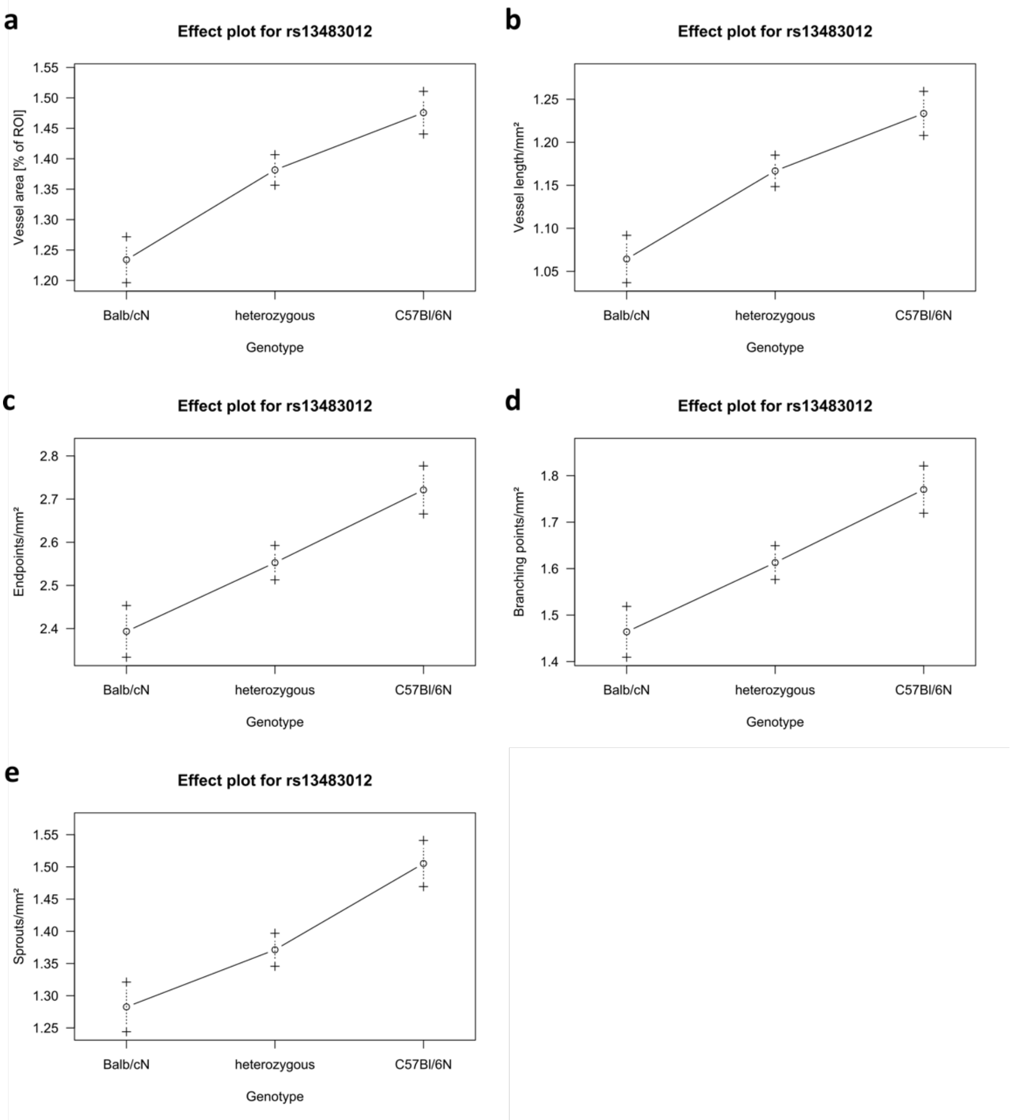

Supplementary Figure 2: Locus effect plots for the reported transformed phenotypes

Marker with the maximum logarithm of the odds (LOD) score on chromosome 17, *rs13483012*, all showing recessive BALB/cN alleles. **A:** Vessel area/mm^2. **B:** Vessel length/mm^2. **C:** End points/mm^2. **D:** Branching points/mm^2. **E:** Sprouts/mm^2.

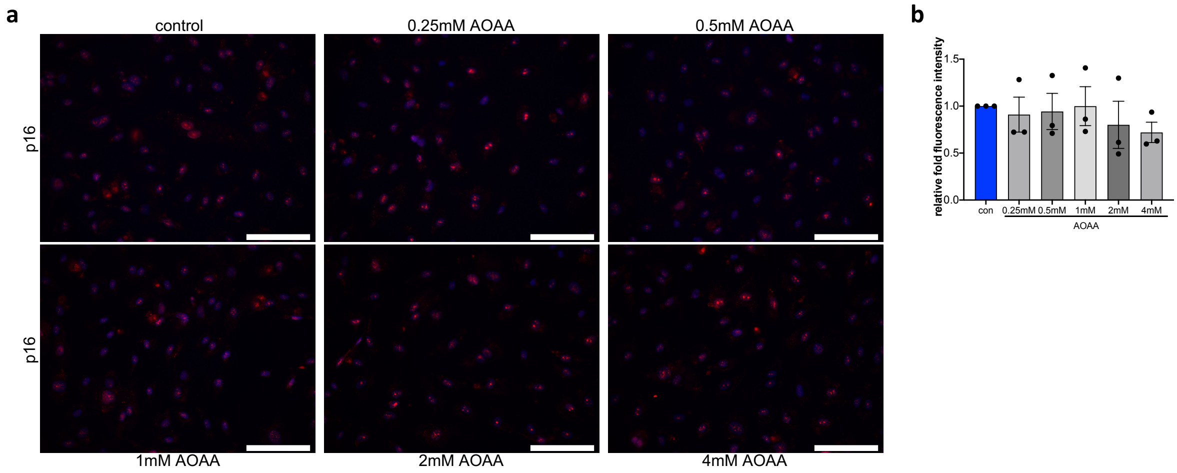

Supplementary Figure 3: Effect of CBS inhibition by AOAA or CBS gene silencing on p16^{INK4A} expression

(**a**) Representative immunofluorescence images of HDLECs treated with indicated concentration of AOAA stained for p16^{INK4A}. Scale bars: 100 µm (**b**) Quantification of immunofluorescence staining of with indicated concentration of AOAA stained for p16^{INK4A}. Data are presented as means ± SEM. Statistical significance was analyzed with one-way ANOVA and Dunnett's multiple comparison test (n=3).

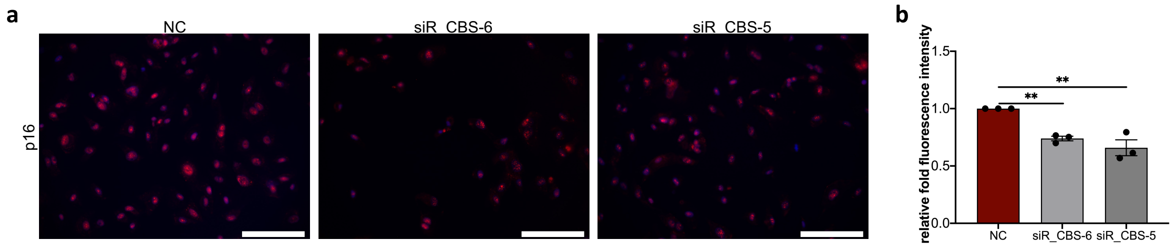

Supplementary Figure 4: Silencing CBS reduces p16^{INK4A} expression

(**a**) Representative immunofluorescence images of HDLECs transfected with with either siR_CBS-5, siR_CBS-6, or negative control (NC) siRNA stained for p16^{INK4A}. Scale bars: 100 µm. (**b**) Quantification of immunofluorescence staining of HDLECs transfected with either siR_CBS-5, siR_CBS-6, or negative control (NC) siRNA stained for p16^{INK4A}. Data are presented as means ± SEM. Statistical significance was analyzed with one-way ANOVA and Dunnett's multiple comparison test (n=3).

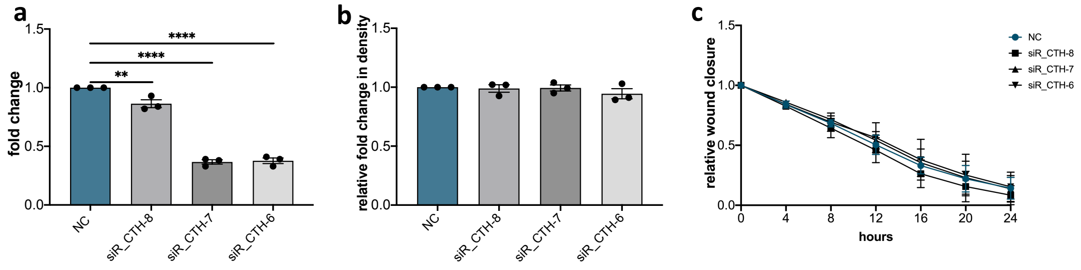

Supplementary Figure 5: Silencing of CTH in HDLECS did not affect proliferation and migration

(**a**) Efficiency of CTH knockdown determined qRT-PCR 72h post-transfection. Reduced expression of CTH in HDLECs after 72h transfected with either siR_CTH-8, siR_CTH-7, siR_CTH-6, or negative control (NC) siRNA (n=3). (**b**) Effect of CTH silencing on HDLECs proliferation transfected with either siR_CTH-8, siR_CTH-7 or siR_CTH-6 compared with NC siRNA transfected cells. Proliferation was determined 72h after transfection by using IncuCyte Zoom (n=3). (**c**) Wound healing assay with negative control (NC) siRNA, siR_CTH-8, siR_CTH-7 or siR_CTH-6 treated HDLECs after 72h transfection (n=3). Data are presented as means ± SEM. Statistical significance was analyzed with (a-b) one-way ANOVA and Dunnett's multiple comparison test or (c) two-way ANOVA and Tukey's multiple comparison test. **p<0.01; ****p < 0.0001.

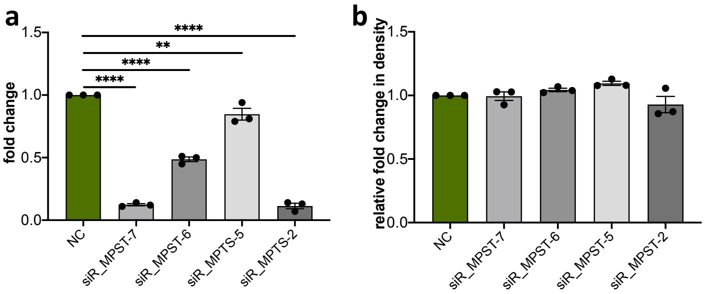

Supplementary Figure 6: Silencing of MPST in HDLECS did not affect proliferation

(**a**) Efficiency of MPST knockdown determined qRT-PCR 72h post-transfection. Reduced expression of MPST in HDLECs after 72h transfected with either siR_MPST-7, siR_MPST-6, siR_MPST-5, siR_MPST-2 or negative control (NC) siRNA (n=3). (**b**) Effect of CTH silencing on HDLECs proliferation transfected with either siR_MPST-7, siR_MPST-6, siR_MPST-5, or siR_MPST-2 compared with NC siRNA transfected cells. Proliferation was determined 72h after transfection by using IncuCyte Zoom (n=3). Data are presented as means ± SEM. Statistical significance was analyzed with one-way ANOVA and Dunnett's multiple comparison test. **p<0.01; ****p < 0.0001.

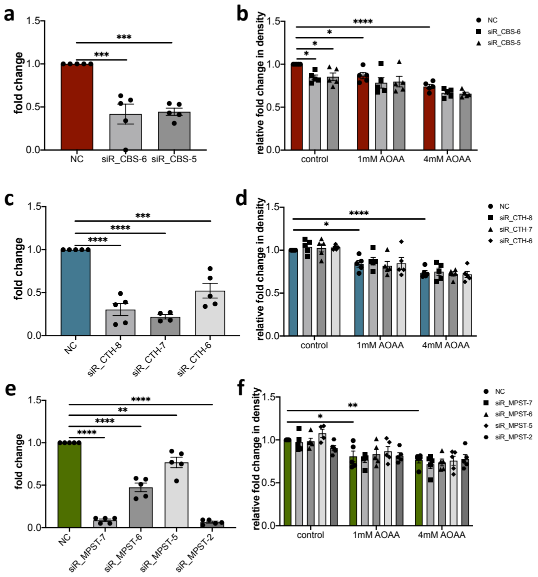

Supplementary Figure 7: **Additional treatment of HDLECs with AOAA after either silencing CBS, CTH or MPST did not influence the proliferation of these cells**

(**a**) Efficiency of CBS knockdown determined qRT-PCR 72h post-transfection. Reduced expression of CBS in HDLECs after 72h transfected with either siR_CBS-5, siR_CBS-6, or negative control (NC) siRNA (n=5). (**b**) Effect of 1mM and 4mM AOAA on HDLECs proliferation transfected with either siR_CBS-5, siR_CBS-6, or negative control (NC) siRNA. Proliferation was determined 72h after transfection by using IncuCyte Zoom (n=5). (**c**) Efficiency of CTH knockdown determined qRT-PCR 72h post-transfection. Reduced expression of CTH in HDLECs after 72h transfected with either siR_CTH-8, siR_CTH-7, siR_CTH-6, or negative control (NC) siRNA (n=5). (**d**) Effect of 1mM and 4mM AOAA on HDLECs proliferation transfected with either siR_CTH-8, siR_CTH-7 or siR_CTH-6 compared with NC siRNA transfected cells. Proliferation was determined 72h after transfection by using IncuCyte Zoom (n=5). (**e**) Efficiency of MPST knockdown determined qRT-PCR 72h post-transfection. Reduced expression of MPST in HDLECs after 72h transfected with either siR_MPST-7, siR_MPST-6, siR_MPST-5, siR_MPST-2 or negative control (NC) siRNA (n=5). (**f**) Effect of 1mM and 4mM AOAA on HDLECs proliferation transfected with either siR_MPST-7, siR_MPST-6, siR_MPST-5, or siR_MPST-2 compared with NC siRNA transfected cells. Proliferation was determined 72h after transfection by using IncuCyte Zoom (n=5). Data are presented as means ± SEM.Statistical significance was analyzed with (a, c, e) one-way ANOVA and Tukey's multiple comparison test or (b, d, f) two-way ANOVA and Tukey's multiple comparison test. *p<0.05 **p<0.01; ***p < 0.001; ****p < 0.0001.

Supplementary Table 1: Raw counts table of F0 animals

Sample ID	Mouse Strain	Replicate	Raw read pairs	Reads pairs after filtering and trimming	Read pairs aligned by Tophat2	Concordant pair alignment rate to Pseudogenome (Tophat2)	Concordant pair alignment rate to mm10 for comparison	Read pairs counted exonic (Htseq)
175101	Balb/cN	1	90.343.042	79.404.780	70.078.832	85,2%	82,2%	40.283.517
175102	Balb/cN	2	89.083.390	79.928.879	70.688.183	85,9%	82,7%	41.152.725
175106	Balb/cN	3	83.456.407	73.521.405	64.340.105	84,6%	81,4%	38.766.069
175107	Balb/cN	4	79.487.806	69.808.817	61.027.616	84,5%	81,4%	36.706.454
175108	C57Bl/6N	1	85.613.684	75.659.511	59.779.387	76,6%	76,6%	35.838.482
175109	C57Bl/6N	2	85.208.546	75.951.107	61.452.665	78,6%	78,5%	36.980.562
175110	C57Bl/6N	3	82.916.298	73.326.353	52.840.989	70,4%	70,3%	29.682.341
175111	C57Bl/6N	4	87.412.337	78.055.095	62.444.083	77,9%	77,8%	36.313.217

Supplementary Data 1: Differential expression analysis on the gene level
