# Supplementary material for: Cystathionine β-synthase as novel endogenous regulator of lymphangiogenesis via modulating VEGF receptor 2 and 3
Source: Commun Biol. 2022 Sep 10;5:950. doi: 10.1038/s42003-022-03923-7 (PMC9464209; doi:10.1038/s42003-022-03923-7)
Supplement: Supplementary file 2 — Description of Additional Supplementary Files [file 42003_2022_3923_MOESM2_ESM.pdf]

Description of Additional Supplementary Files

File name: Supplementary Data 1
Description: Differential expression analysis on the gene level.

File name: Supplementary Data 2
Description: Raw data underlying data shown in main figures.
