# Supplementary material for: Cystathionine β-synthase as novel endogenous regulator of lymphangiogenesis via modulating VEGF receptor 2 and 3
Source: Commun Biol. 2022 Sep 10;5:950. doi: 10.1038/s42003-022-03923-7 (PMC9464209; doi:10.1038/s42003-022-03923-7)
Supplement: Supplementary file 5 — Reporting Summary [file 42003_2022_3923_MOESM5_ESM.pdf]

nature portfolio

Corresponding author(s): Dr. Thomas Clahsen

Last updated by author(s): 2022.07.14

Reporting Summary

Nature Portfolio wishes to improve the reproducibility of the work that we publish. This form provides structure for consistency and transparency in reporting. For further information on Nature Portfolio policies, see our Editorial Policies and the Editorial Policy Checklist.

Statistics

For all statistical analyses, confirm that the following items are present in the figure legend, table legend, main text, or Methods section.

n/a	Confirmed	
☐	☒	The exact sample size (*n*) for each experimental group/condition, given as a discrete number and unit of measurement
☐	☒	A statement on whether measurements were taken from distinct samples or whether the same sample was measured repeatedly
☐	☒	The statistical test(s) used AND whether they are one- or two-sided<br>*Only common tests should be described solely by name; describe more complex techniques in the Methods section.*
☒	☐	A description of all covariates tested
☐	☒	A description of any assumptions or corrections, such as tests of normality and adjustment for multiple comparisons
☐	☒	A full description of the statistical parameters including central tendency (e.g. means) or other basic estimates (e.g. regression coefficient) AND variation (e.g. standard deviation) or associated estimates of uncertainty (e.g. confidence intervals)
☐	☒	For null hypothesis testing, the test statistic (e.g. *F*, *t*, *r*) with confidence intervals, effect sizes, degrees of freedom and *P* value noted<br>*Give P values as exact values whenever suitable.*
☒	☐	For Bayesian analysis, information on the choice of priors and Markov chain Monte Carlo settings
☒	☐	For hierarchical and complex designs, identification of the appropriate level for tests and full reporting of outcomes
☒	☐	Estimates of effect sizes (e.g. Cohen's *d*, Pearson's *r*), indicating how they were calculated

Our web collection on statistics for biologists contains articles on many of the points above.

Software and code

Policy information about availability of computer code

Data collection	Cellsens Dimension 1.8 & 3.1<br>FACS DIVA 8.0.2<br>IncuCyte Zoom 2016B<br>CFX Manager 2.1 & CFX Maestro 1.1<br>QuantStudioTM 6 and 7 Flex Real-Time PCR System Software

Data analysis	GraphPad Prism 8.4.3<br>FlowJo 8.7.3<br>IncuCyte Zoom 2016B<br>Cell^F<br>cutadapt (version 1.10)<br>Sanger mouse genomes project (Release 1505)<br>GRCm38 reference genome using modtools (version 1.0.2)<br>Tophat2 (version 2.1.1)<br>Lapels (version 1.1.1)<br>Ensembl mouse gene model (Release 94) with HTSeq (version 0.7.1)<br>DESeq2 (version 1.30)<br>fdrtool (version 1.2.16)<br>Matlab_R2021a<br>QuantStudioTM 6 and 7 Flex Real-Time PCR System Software<br>Design & Analysis Software 2.6.0. Real-time PCR System Applied Biosystems

For manuscripts utilizing custom algorithms or software that are central to the research but not yet described in published literature, software must be made available to editors and reviewers. We strongly encourage code deposition in a community repository (e.g. GitHub). See the Nature Portfolio guidelines for submitting code & software for further information.

Data

Policy information about availability of data

All manuscripts must include a data availability statement. This statement should provide the following information, where applicable:
- Accession codes, unique identifiers, or web links for publicly available datasets
- A description of any restrictions on data availability
- For clinical datasets or third party data, please ensure that the statement adheres to our policy

Sequencing data and normalized expression values were uploaded to the Gene Expression Omnibus at GSE178730.

Human research participants

Policy information about studies involving human research participants and Sex and Gender in Research.

Reporting on sex and gender	non-human research study
Population characteristics	not applicable
Recruitment	not applicable
Ethics oversight	not applicable

Note that full information on the approval of the study protocol must also be provided in the manuscript.

Field-specific reporting

Please select the one below that is the best fit for your research. If you are not sure, read the appropriate sections before making your selection.

☒ Life sciences ☐ Behavioural & social sciences ☐ Ecological, evolutionary & environmental sciences

For a reference copy of the document with all sections, see nature.com/documents/nr-reporting-summary-flat.pdf

Life sciences study design

All studies must disclose on these points even when the disclosure is negative.

Sample size	Primary cells were used and thus required no sample size calculation. In all in vivo experiments at least 6 animals per group was used to perform stastistical analysis. No sample-size calculation was necessary or performed since this study is of pre-clinical development stage.
Data exclusions	To detect an outlier, the ROUT methods was used.
Replication	In the individual in vitro experiments, technical replicates were used, which already reflects the reproducibility in the individual experiment. All replication attempts were successful.
Randomization	This is not relevant to ous study.
Blinding	Since this study is of pre-clinical development stage, blinding was not necessary or used in this study.

Reporting for specific materials, systems and methods

We require information from authors about some types of materials, experimental systems and methods used in many studies. Here, indicate whether each material, system or method listed is relevant to your study. If you are not sure if a list item applies to your research, read the appropriate section before selecting a response.

Materials & experimental systems

n/a	Involved in the study	
☐	☒	Antibodies
☐	☒	Eukaryotic cell lines
☒	☐	Palaeontology and archaeology
☐	☒	Animals and other organisms
☒	☐	Clinical data
☒	☐	Dual use research of concern

Methods

n/a	Involved in the study	
☒	☐	ChIP-seq
☐	☒	Flow cytometry
☒	☐	MRI-based neuroimaging

Antibodies

Antibodies used	rabbit anti-mouse LYVE-1 antibody (1:300 dilution; Cat No. 11-034, AngioBio Co., Del Mar, CA)<br>CD31 Clone ER-MP12 (1:200 dilution; Cat No. BM4086, OriGene Technologies GmbH, Herford, Germany)<br>F4/80-AlexaFluor647 (Clone BM3) (1:100, BioLegend, Cat. No. 123122, BioLegend, Koblenz, Germany)<br>goat anti-rabbit AlexaFluor 488 (1:500 dilution; Cat No. A11008 Invitrogen, Darmstadt, Germany)<br>goat anti-rat AlexaFluor 555 (1:500 dilution; Cat No. A21434 Invitrogen, Darmstadt, Germany)<br>CBS Clone B-4 (1:50 dilution; Cat No., sc133154, Santa Cruz, Heidelberg, Germany)<br>p16 Clone EPR1473 (1:100 dilution; Cat No. ab108349, Abcam, Amsterdam, Netherlands)<br>goat anti-mouse AlexaFluor 488 (1:100 dilution; Cat No. A21121 Invitrogen, Darmstadt, Germany)<br>goat anti-rabbit Cy3 (1:100 dilution; Cat No. 111-165-045, Dianova, Hamburg, Germany)
Validation	All antibodies were were validated, datasheets are available, and we followed manufactors's instruction

Eukaryotic cell lines

Policy information about cell lines and Sex and Gender in Research

Cell line source(s)	primary human dermal lymphatic endothelial cells
Authentication	Cell were purchased from Promocell (Heidelberg)
Mycoplasma contamination	The human dermal lymphphatic endothelial cell were tested for mycoplasm with the MycVenor GeM Mycoplasmen-Diagnosis kit (Minerva Biolabs)
Commonly misidentified lines<br>(See ICLAC register)	No misidentified cell lines used

Animals and other research organisms

Policy information about studies involving animals; ARRIVE guidelines recommended for reporting animal research, and Sex and Gender in Research

Laboratory animals	Male C57BL/6N and female or male BALB/cN mice, aged between 8 and 10 weeks;<br>Mice used for the suture-induced inflammatory corneal neovascularization assay were 6- to 10-week-old female C57BL/6NCrl or BALB/cAnNCrl mice.
Wild animals	This study did not involve wild animal.
Reporting on sex	The sex of the animals does not influence the research question or the target formation. However, for better comparability with previously obtained findings only female animals were used.
Field-collected samples	This study did not involve samples collected from the field.
Ethics oversight	The experiments were approved by the local animal care committee LANUV North Rhine-Westphalia (AZ 84-02.05.2011.210 and AZ 81-02.04.2020.A199) and are in accordance with institutional and national guidelines.

Note that full information on the approval of the study protocol must also be provided in the manuscript.

March 2021

Flow Cytometry

Plots

Confirm that:

☒ The axis labels state the marker and fluorochrome used (e.g. CD4-FITC).

☒ The axis scales are clearly visible. Include numbers along axes only for bottom left plot of group (a 'group' is an analysis of identical markers).

☒ All plots are contour plots with outliers or pseudocolor plots.

☒ A numerical value for number of cells or percentage (with statistics) is provided.

Methodology

Sample preparation	For the apoptosis assays the BioLegend FITC Annexin V apoptosis detection kit with manufacturer. HDLEC were seeded onto a 6-well plate at a cell density of 1.25x105/well in complete endothelial cell growth medium MV2 (PromoCell, Heidelberg, Germany). On the next day, the cells were incubated in basal medium supplemented with 1% FCS (minimal medium) for 1h. Following treatment of HDLECs with different concentrations of AOAA (0.25mM, 0.5mM, 1mM, 2mM, 4mM) for 24h, cells were harvested, stained according manufactors instruction.<br>HDLEC were seeded onto a 6-well plate at a cell density of 1.25x105/well in complete endothelial cell growth medium MV2 (PromoCell, Heidelberg, Germany). On the next day, the cells were transfected with CBS specific siRNA (siR_CBS-6 or siR_CBS-5) or non specific siRNA NC control. 72h after transfection cells were harvested, stained according manufactors instruction.
Instrument	Bioscience FACS Canto II
Software	data collection: FACS DIVA 8.0.2<br>data evaluation: FlowJo 8.7.2
Cell population abundance	The cells used are commercially acquired pure primary lymphatic endothelial cells. The percentage of cells in the FSC/SSC gate is min. 85%.
Gating strategy	1. FSC/SSC -> 2. doublet discrimination -> 3. Annixin V-FITC / 7AAD

☐ Tick this box to confirm that a figure exemplifying the gating strategy is provided in the Supplementary Information.
